# TWISP: a transgenic worm for interrogating signal propagation in *Caenorhabditis elegans*

**DOI:** 10.1093/genetics/iyae077

**Published:** 2024-05-11

**Authors:** Anuj Kumar Sharma, Francesco Randi, Sandeep Kumar, Sophie Dvali, Andrew M Leifer

**Affiliations:** Department of Physics, Princeton University, Princeton, NJ 08544, USA; Department of Physics, Princeton University, Princeton, NJ 08544, USA; Princeton Neuroscience Institute, Princeton University, Princeton, NJ 08544, USA; Department of Physics, Princeton University, Princeton, NJ 08544, USA; Department of Physics, Princeton University, Princeton, NJ 08544, USA; Princeton Neuroscience Institute, Princeton University, Princeton, NJ 08544, USA

**Keywords:** functional connectivity, calcium imaging, optogenetics, GUR-3, PRDX-2, dexamethasone, drug-inducible gene expression, *Caenorhabditis elegans*, neurons

## Abstract

Genetically encoded optical indicators and actuators of neural activity allow for all-optical investigations of signaling in the nervous system. But commonly used indicators, actuators, and expression strategies are poorly suited for systematic measurements of signal propagation at brain scale and cellular resolution. Large-scale measurements of the brain require indicators and actuators with compatible excitation spectra to avoid optical crosstalk. They must be highly expressed in every neuron but at the same time avoid lethality and permit the animal to reach adulthood. Their expression must also be compatible with additional fluorescent labels to locate and identify neurons, such as those in the NeuroPAL cell identification system. We present TWISP, a transgenic worm for interrogating signal propagation, that addresses these needs and enables optical measurements of evoked calcium activity at brain scale and cellular resolution in the nervous system of the nematode *Caenorhabditis elegans*. In every neuron we express a nonconventional optical actuator, the gustatory receptor homolog GUR-3 + PRDX-2, under the control of a drug-inducible system QF + hGR, and a calcium indicator GCAMP6s, in a background with additional fluorophores from the NeuroPAL cell ID system. We show that this combination, but not others tested, avoids optical crosstalk, creates strong expression in the adult, and generates stable transgenic lines for systematic measurements of signal propagation in the worm brain.

## Introduction

A fundamental goal of neuroscience is to understand how neural signals flow through the brain to process information and generate actions. Genetic model systems are crucial for this understanding, in part, because they provide a platform to express genetically encoded optical indicators and actuators for measuring and manipulating neural activity (Simpson and Looger 2018; Randi and Leifer 2020; Bergs *et al*. 2022). Genetically encoded calcium or voltage indicators combined with light-gated ion channels have enabled all-optical functional investigations of neural signaling in circuits or sub-brain regions (Guo *et al*. 2009; Rickgauer *et al*. 2014; Emiliani *et al*. 2015; Franconville *et al*. 2018). Now there is interest in performing such investigations at nervous system scale.

The *Caenorhabditis elegans* (*C. elegans*) nervous system already has a well-mapped anatomical wiring diagram, called a connectome (White *et al*. 1986; Cook *et al*. 2019; Witvliet *et al*. 2021) and a cell-resolved atlas of gene expression (Taylor *et al*. 2021). Adding measurements of how neural signals propagate at nervous system scale and cellular resolution can help reveal how this nervous system's known structure and gene expression relates to its function. For example, signal propagation measurements can help clarify the role of extrasynaptic peptidergic signaling between neurons in worms (Beets *et al*. 2023; Ripoll-Sanchez *et al*. 2023) and in mammals (Smith *et al*. 2019) which is not visible from the wiring diagram. A major challenge to performing these measurements, however, is that commonly used combinations of indicators, actuators, and expression strategies are poorly suited for measuring neural signal propagation at nervous system scale. Many approaches suffer from unwanted neural activation during imaging due to spectral overlap. For example, 488 nm light typically used to image calcium activity with GCaMP (Chen *et al*. 2013) will activate Chrimson at ∼35% of its on-peak photocurrents (Klapoetke *et al*. 2014). Driving broad expression is challenging generally. To our knowledge, light-gated ion channel expression has not been previously reported in every single neuron in a brain, possibly because, as we discover here, high expression of optogenetic actuators throughout the brain can be toxic and sometimes lethal.

An indicator and actuator pair are needed that: (1) avoid spectral overlap, so that neural activity can be imaged without unwanted neural activation; (2) can be expressed at sufficiently high levels to allow robust measurements while still avoiding lethality; and (3) are compatible with fluorescent reporters to identify each neuron with respect to the connectome (Yemini *et al*. 2021).

In this work, we tested various combinations of calcium indicator, neural actuators, and expression strategies in the nematode *C. elegans*. We present TWISP, a Transgenic Worm for Interrogating Signal Propagation, and demonstrate its suitability for large-scale signal propagation mapping of the brain.

## Material and methods

### Molecular cloning and plasmids

Plasmids generated in this study are listed in Supplementary Table 2. We used a seamless cloning strategy (In-Fusion, Takara Bio USA, Inc.). Primers were synthesized from the company IDT. Clones were confirmed by Sanger sequencing (AZENTA Life Sciences, USA). In brief, we first PCR amplified both back bones and inserts using PrimeSTAR GXL DNA Polymerase, a hot start, high-fidelity polymerase (Cat#R050A, Takara Bio USA, Inc.) as per manufacturer's instructions. Both back bones and inserts were then agarose-gel purified using NucleoSpin Gel and PCR Clean-Up columns (Takara Bio USA, Inc.). Purified fragments were then mixed at prescribed molar ratio together with In-Fusion HD Cloning Kit (Cat#639649, Takara Bio USA, Inc.), and incubated at 50°C for 15 min. A total of 2 µl of each reaction was then transformed into 100 µl Stellar competent cells as per manufacturer's instructions and plated on LB plates containing appropriate selectable antibiotics.

### Worm maintenance

*C. elegans* strains were maintained according to procedures described in Brenner (1974) with slight modifications. All worms were handled and maintained in near dark condition, at 20°C. Worms were exposed to dim brightfield light during transferring. All worm strains used in this study are listed in Supplementary Table 1.

### Transgenic strains

We used micro-injections for generating transient transgenic lines followed by UV integration and miniSOG mediated rapid integration methods to create integrated transgenic animals as needed (Evans 2006; Noma and Jin 2018). Detailed information regarding plasmid concentrations injected for each transgenic worm is provided in Supplementary Table 1. Worms created for this study will be made available from Caenorhabditis Genetics Center (CGC), University of Minnesota.

### All-trans retinal (ATR) and dexamethasone (dex) treatment

Worms that expressed optogenetic actuators from the rhodopsin family, such as eTsChR, were cultivated on plates containing the necessary co-factor all-trans retinal (ATR). To prepare ATR plates, we seeded an NGM plate with 250 µl *Escherichia coli*  OP50 culture mixed with 1.25 µl of 100 mM ATR from stock, a day prior to treatment. ATR stocks (100 mM) were prepared by dissolving 100 mg of ATR (Cat# R2500, Sigma-Aldrich) in 3.52 ml Ethyl alcohol and then filter-sterilized using 0.2 µm filters. ATR stocks were then aliquoted and stored in smaller volume at −20°C, in dark tubes.

Animals that express actuators under the control of the drug-inducible QF + hGR> QUAS system were treated with dexamethasone (dex) to induce gene expression prior to experiments, typically for the approximately 20 h prior to young adulthood. To prepare Dex-NGM plates, we added 2 ml of dex stock solution (100 mM dex in DMSO) to each liter of NGM-agar media, 5 min before pouring the plates, while stirring. Dex-plates were stored at 4°C for up to a month. Dex stocks were prepared by dissolving 1 g Dexamethasone (Cat# D1756, Sigma-Aldrich) in 25.5 ml DMSO (Cat#D8418, Sigma-Aldrich). Dex stocks were filter sterilized, aliquoted, and stored at −80°C in the dark until needed.

### Light-evoked behavior response assay

To test the behavioral response of transgenic worms expressing various pan-neuronal actuators, we scored the animal's behavior in response to illumination in a fluorescent stereoscope (Leica-M205FA, Leica Microsystems). Young adult animals were illuminated with either 475 nm or 505 nm light. For 475 nm light, the microscope's built in fluorescent excitation source was used (Supplementary Fig. 1 shows spectra measured via a portable spectrometer). For 505 nm light, a custom built external LED (M505L4, Thorlabs) was used. Light intensities were adjusted such that the worm was illuminated with ∼1.5 mW/mm^2^ of light as measured by a power meter placed at the focal plane. Worms were illuminated for 10–20 s and their behavior response was scored manually using the 4-point scoring criteria described in the main text.

### Multi-generation growth assay

For the multi-generation growth assay described in Fig. 4, observations were recorded about the animals’ health and stage over a series of days. In brief, two L4 worms were transferred to either regular- or Dex-NGM plates seeded with *E. coli*  OP50, three plates each trial. After three days, plates were evaluated for the presence of the most advanced stage achieved by any progeny on the plate. If needed (in case of dex-treatment), plates were further evaluated on day 4 and day 5 for the presence of L4 animals among progeny. We further transferred two-L4 worms from dex-treatment, to new regular (to recover) and dex (to continue treatment) NGM plates in the second round and evaluated the plates for progeny growth after three days. The stage of the animal that reached the latest developmental stage is reported for the animals in Fig. 4. So, for example, if most animals reached L3 but one animal reached L4, L4 is reported.

### Growth and progeny production assay

For measuring growth and progeny production rate in Fig. 5, we recorded the proportion of animals that reached adulthood in a given time starting from the egg stage, and calculated the progeny produced using a semi-synchronization method as adapted from Kim *et al*. (2018). Briefly, three adult hermaphrodites were placed on an NGM plate seeded with *E. coli*  OP50 and allowed to lay eggs for 3 h. Adult worms were then removed, and the eggs were allowed to grow in the dark, at 20°C to obtain age-synchronized animals. The total number of progeny and percentage of worms that reached adulthood were counted at 70 h (when all WT N2 progeny typically reach the adult stage) and at 94 h (when roughly half of AML462's progeny reached adulthood). Progeny production rate per animal was calculated by dividing the number of progeny on the plate at the end of the assay by the original number of adults that started on the plate and dividing by the number of hours elapsed. The number of plates used is reported in Supplementary Tables 3, 4, and 5.

### Locomotion measurements

A high-throughput automated behavior imaging system was used to quantify attributes of the animal's locomotion (Liu *et al*. 2018; Liu *et al*. 2022). Age-synchronized animals were obtained by bleaching gravid animals. Eggs were then left on a shaker at 450 rpm, overnight. The next day, the L1 larvae were placed on *E. coli*  OP50 seeded NGM plates and stored in the dark at 20°C. Once the worms reached day 1 young adult stage, size and locomotion were measured as in Liu *et al*. (2022). Dex treated worms were first transferred to 200 µM dex-containing NGM plates ∼20 h prior to measurements.

Locomotion for each strain was measured on at least four plates with typically 30–40 animals per plate. Unlike other assays which report a single metric per plate, and then average across plates, here we calculate a single behavior metric for each worm track and then average across tracks comingled from all plates. Each track corresponds to a single worm, although one worm typically will have multiple tracks because tracks stop and start when a worm wanders out of the field of view and returns, or collides with another worm, as described in Liu *et al*. (2018), Liu *et al*. (2022), and Kumar *et al*. (2023). We sorted tracks on the basis of distance traveled from largest to smallest for each strain and then picked five worms (one median and two from each side) as representative for Supplementary Fig. 3.

### Gentle-touch behavior assay

We used a manual gentle-touch assay with an eyelash with some modification (Chalfie *et al*. 2014). Briefly, six young adult worms were picked to a new unseeded NGM plate. Worms were allowed to move freely for 5 min before testing for response to eyelash touches. Thereafter, each worm was tested by touching only the anterior half, three times at 1 min intervals with a human eyelash. Behavior in response to touch was observed and scored as either: a reversal ending in turn, a reversal only, or no response.

### Signal propagation and calcium imaging measurements

Signal propagation measurements and analysis were performed as described in Randi *et al*. (2023). Whole-brain calcium imaging was performed on immobilized TWISP animals while individual neurons were stimulated, one-at-a-time, every 60 s or 30 s, for 0.5 s via 850 nm 2-photon laser pulses at 500 kHz repetition rate. Before calcium imaging experiments began, multicolor imaging was used to record the color of each neuron for identification via the NeuroPAL system (Yemini *et al*. 2021). After experiments were completed, neurons were segmented based on their tagRFP-T fluorescence and fluorescent calcium traces were extracted, via an automated analysis pipeline. Neuron identities were assigned manually based on each neuron's position and color code from NeuroPAL. Only traces for neurons that were confidentially assigned a neuron identity were included. Animals were immobilized on 10% agarose pads in 6 µl M9 buffer, with 2 µl of 100 nm polystyrene beads (Cat# 00876-15, Polysciences) and 2 µl of levamisole (from 500 µM stock, Cat# 155228, MP Biomedicals). For head and tail recordings, animals were immobilized such that both the head and tail were visible in the field of view.

### Statistical analysis

Graphs are plotted either using Prism-v.10 (GraphPad Software LLC) (Figs. 2a, 3a, and 5) or Matlab (MathWorks) (Fig. 6). Kruskal–Wallis (one-way ANOVA) with Dunn's multiple comparisons test was used for calculating statistical significance in Figs. 5 and 6.

## Results

### GUR-3 + PRDX-2 and TsChR have excitation spectra compatible with GCaMP imaging

**Fig. 1.**
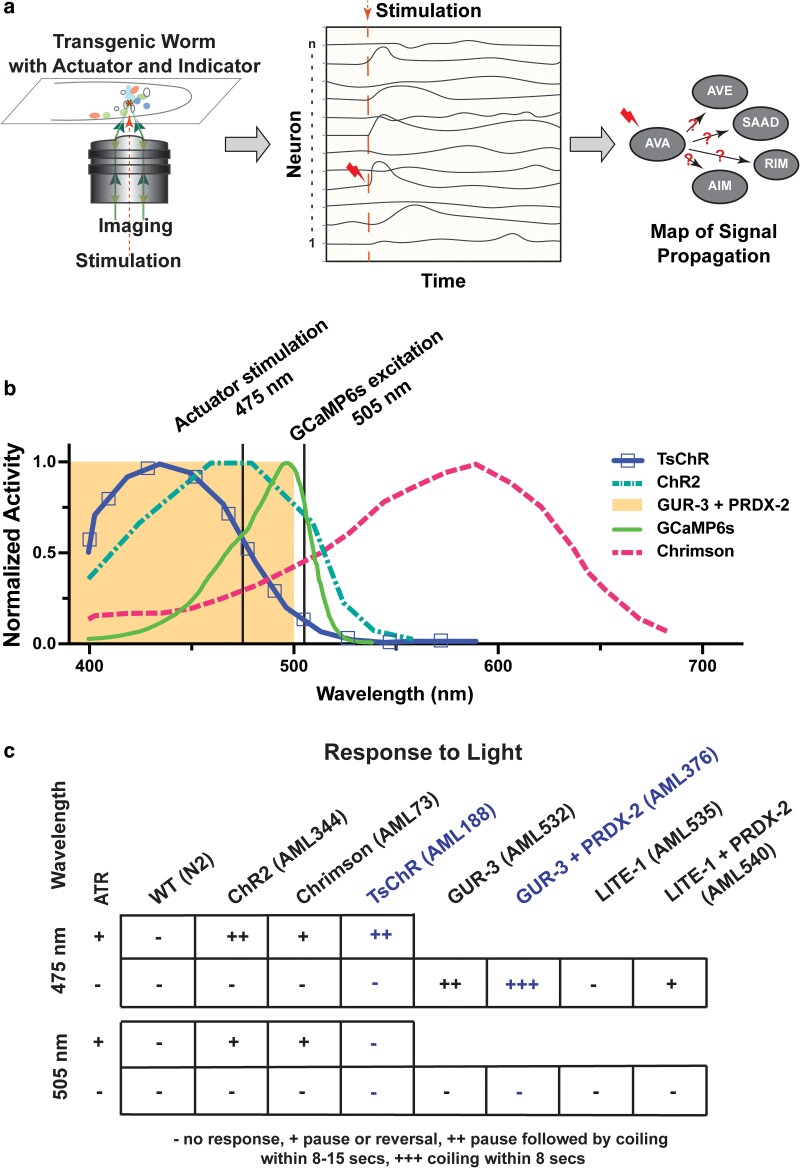
Strategy for all-neuron random-access stimulation and calcium imaging without optical cross talk. a) Schematic of a transgenic *C. elegans* for measuring signal propagation via optogenetic stimulation and calcium imaging. b) Previously reported action spectra for several neural actuators, compared with the absorbance spectra of GCaMP6s. Adapted from: Chen *et al*. (2013), Husson *et al*. (2013), Klapoetke *et al*. (2014), Bhatla and Horvitz (2015), and Dana *et al*. (2016). Shaded area indicates the action spectral range of GUR-3 + PRDX-2. 505 nm light is used to excite GCaMP6s close to its absorbance peak of 498 nm. c) Optogenetic proteins were expressed in every neuron under a *rab-3* promoter. Behavior response to 1.5 mW/mm^2^ illumination of either 505 nm or 475 nm light is shown. For animals expressing rhodopsin-based optogenetic proteins, behavior is measured with and without the necessary co-factor all-trans retinal (ATR). TsChR and GUR-3 + PRDX-2 (highlighted in blue text) show good compatibility with GCaMP6s imaging wavelengths.

We sought a genetically encoded neural activity indicator and optogenetic actuator that could be co-expressed in each cell to provide optical access to measure and manipulate neural activity of all neurons (Fig. 1a). We focused on calcium indicators because recent families of calcium indicators have typically had brighter fluorescence with larger signal-to-noise ratios than commonly used voltage indicators (Azimi Hashemi *et al*. 2019; Abdelfattah *et al*. 2020; Abdelfattah *et al*. 2023). The GCaMP family of calcium indicators is the most widely used (Chen *et al*. 2013) and has a single-photon absorbance peak at approximately 498 nm (Fig. 1b). A challenge of using GCaMP in combination with optogenetic actuators is that wavelengths used to excite GCaMP near its peak also excite common optogenetic proteins including ChR2 and Chrimson (Fig. 1c). For example, Chrimson is reported to be excited to roughly ∼35% of its maximal photocurrents by 488 nm light that is commonly used to image GCaMP (Klapoetke *et al*. 2014). This optical crosstalk leads to unwanted optogenetic stimulation and poses a challenge for single-photon calcium imaging. For example, prior work in *C. elegans* has tried to work around this crosstalk by ignoring all but the first few seconds of imaging, reasoning that the first few seconds may still be interpretable despite the presence of crosstalk because optogenetic activation is typically faster than the calcium response (Lu *et al*. 2022). Two-photon imaging of GCaMP provides alternative strategies for avoiding unwanted optical activation (Rickgauer *et al*. 2014; Emiliani *et al*. 2015; Packer *et al*. 2015), but we specifically sought a single-photon imaging solution because of its speed and relative ease of use (Nguyen *et al*. 2016). Another strategy to avoid spectral overlap with opsins such as ChR2 (Dana *et al*. 2016) would be to use red-shifted calcium indicators, but in our hands, neither jRGECO1a nor jRCaMP1b (Dana *et al*. 2016) was sufficiently bright for the fast volumetric imaging needed here (short 20 ms camera exposures). We therefore explored blue-shifted optogenetic actuators that might work better with GCaMP6s. An actuator that is sufficiently blue shifted should respond to short wavelength light, but not to the longer wavelength light used to image GCaMP6s.

**Fig. 2.**
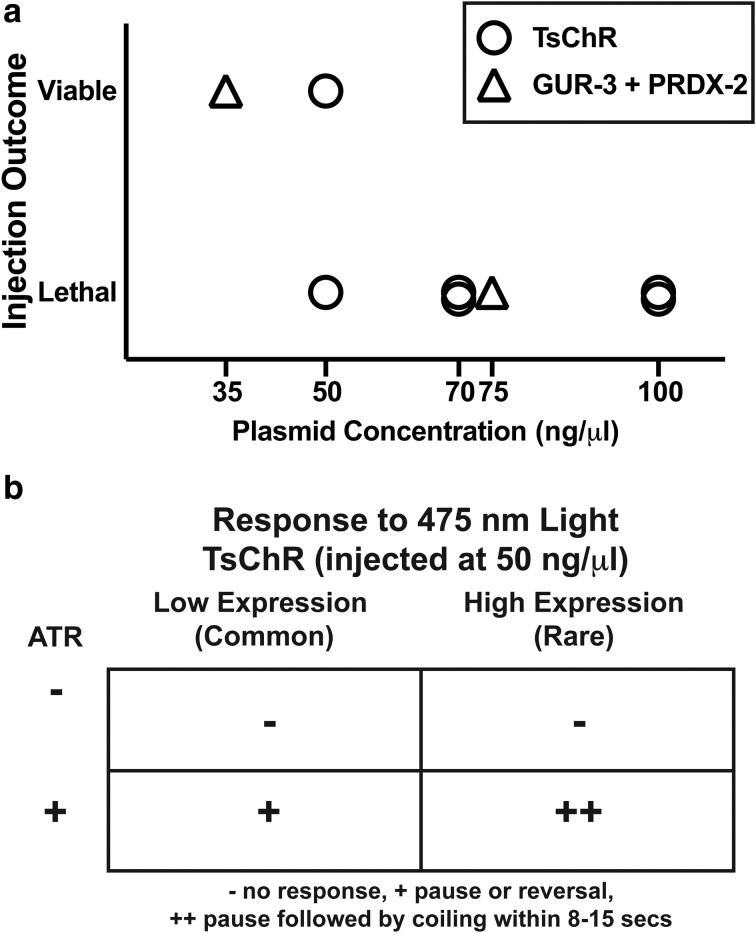
High concentration injections of actuator-containing plasmids are not viable for transgenesis, but higher expression is desirable. a) Injection concentration for plasmids containing either GUR-3 + PRDX-2 or TsChR and the viability of transgene expressing progeny is shown. Each symbol indicates one trial. b) Light-evoked behavior response of worms expressing TsChR (50 ng/µl injection) in all neurons upon 1.5 mW/mm^2^ 475 nm blue-light illumination, with and without the co-factor ATR (all-trans retinal). Most worms had very low expression, as estimated from reporter expression. Rare worms with high expression showed stronger responses.

**Fig. 3.**
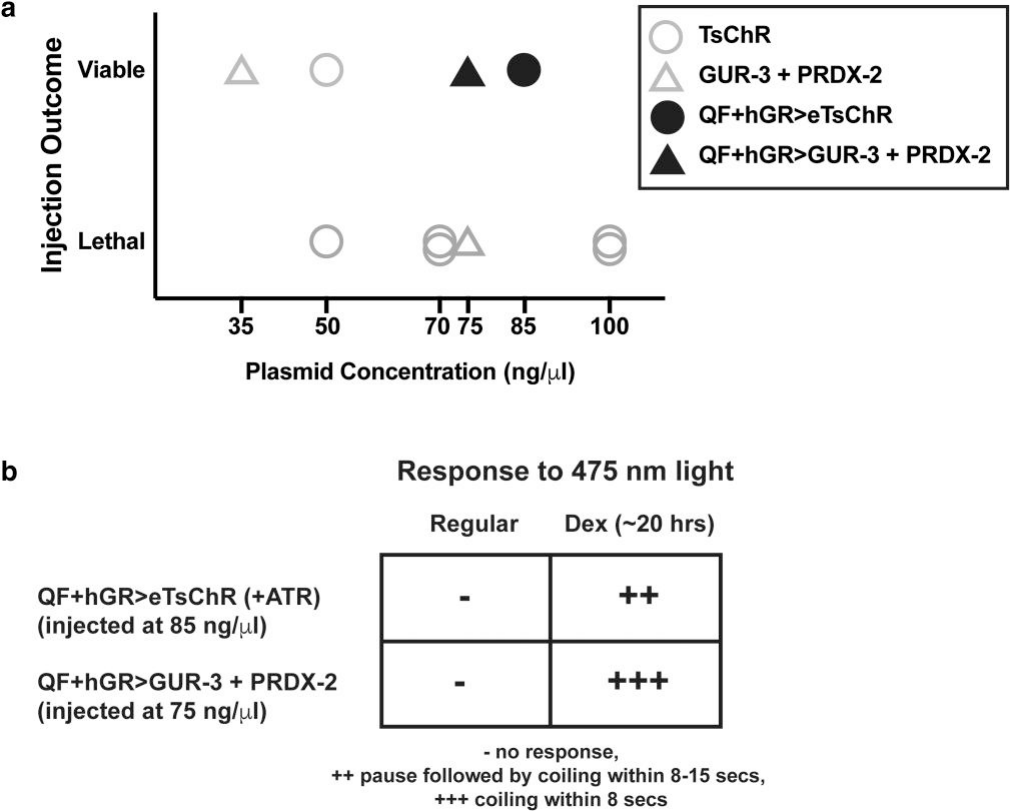
Drug-inducible expression enables robust light response while avoiding lethality a) Injections of plasmids containing actuators under the control of the QF + hGR drug-inducible expression system are viable at higher injection concentrations (black filled shapes) than injections of plasmids for direct expression of the actuators (gray open shapes, same as Fig. 2). b) Exposure to the drug dexamethasone (Dex) evokes actuator expression and confers robust light response to 475 nm illumination.

**Fig. 4.**
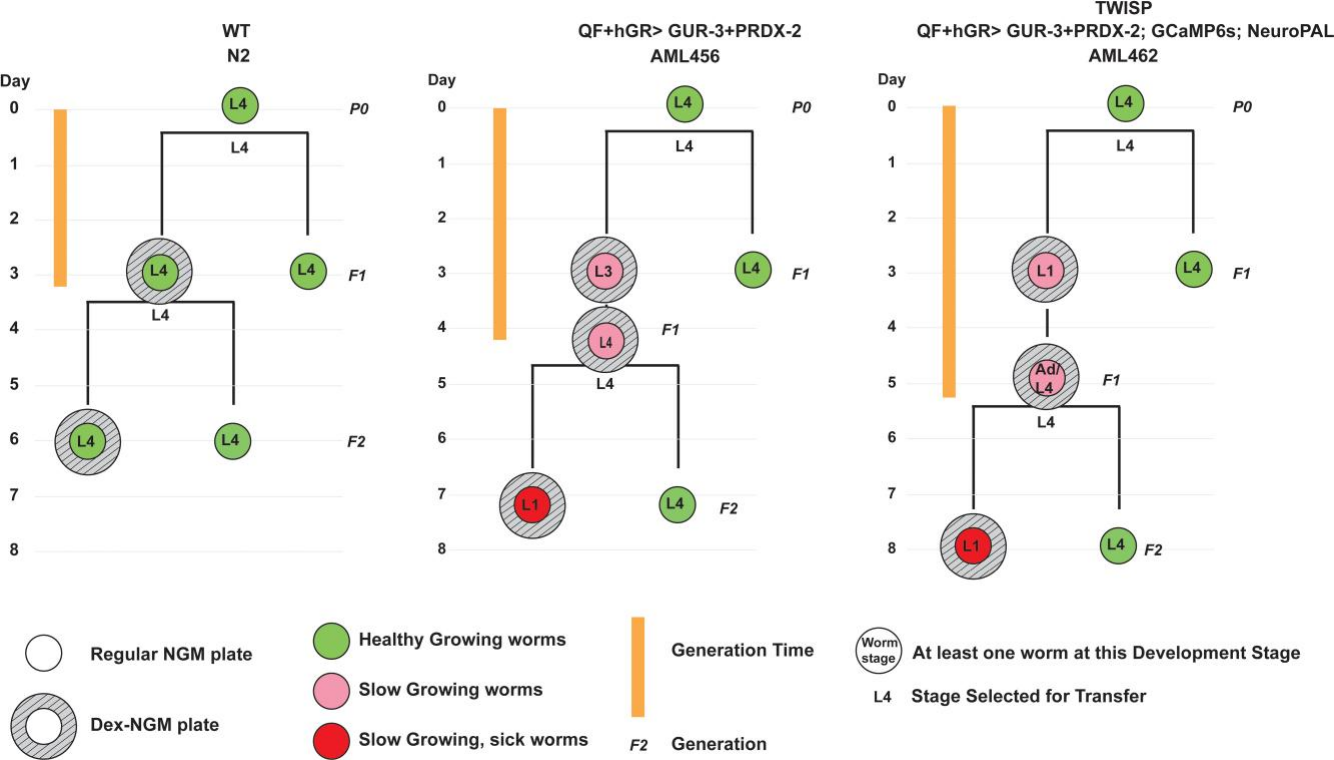
Drug induced actuator expression modulates health and growth. Animals are observed as they are propagated on plates across multiple generations either on or off the drug dexamethasone. Observations of the animal's health and developmental stage are made at time points indicated by the location of the circle on the timeline. For each observation, the developmental stage of the most advance animal found on the plate is reported (listed inside the circle). L4s were always selected for transfer to new plates. Health and generation times improve for the progeny of animals that had previously been exposed to the drug but subsequently cultivated on regular NGM plates.

**Fig. 5.**
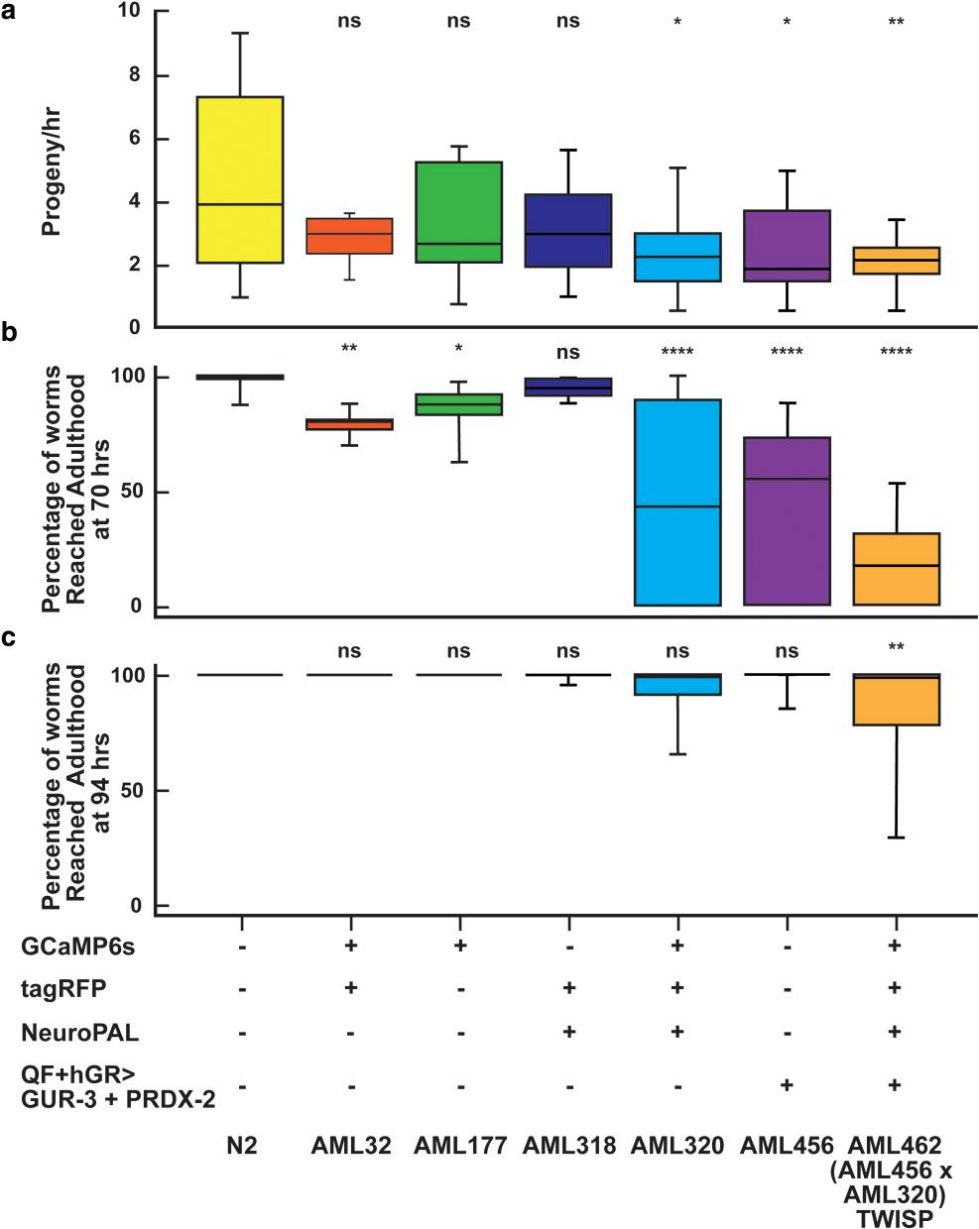
Rate of growth and progeny production decrease with transgenic load, GCaMP6s, NeuroPAL system, and actuator expression. a) Rate of progeny production and b) percentage of animals that reach adulthood in 70 h or c) in 94 h is reported for strains carrying various components of the TWISP system. Box shows median and 25th and 75th percentile values, whiskers show min and max values. Mean ± SD values and number of plates are reported in Supplementary Tables 3, 4, and 5. Experiments were performed in triplicates and repeated at least 3 times for all strain except AML32 and AML177 in (c). Statistical significance is with respect to WT, using Kruskal–Wallis test followed by Dunn's multiple comparisons, *<0.0332, **<0.0021, ***<0.0002, and ****<0.0001.

**Fig. 6.**
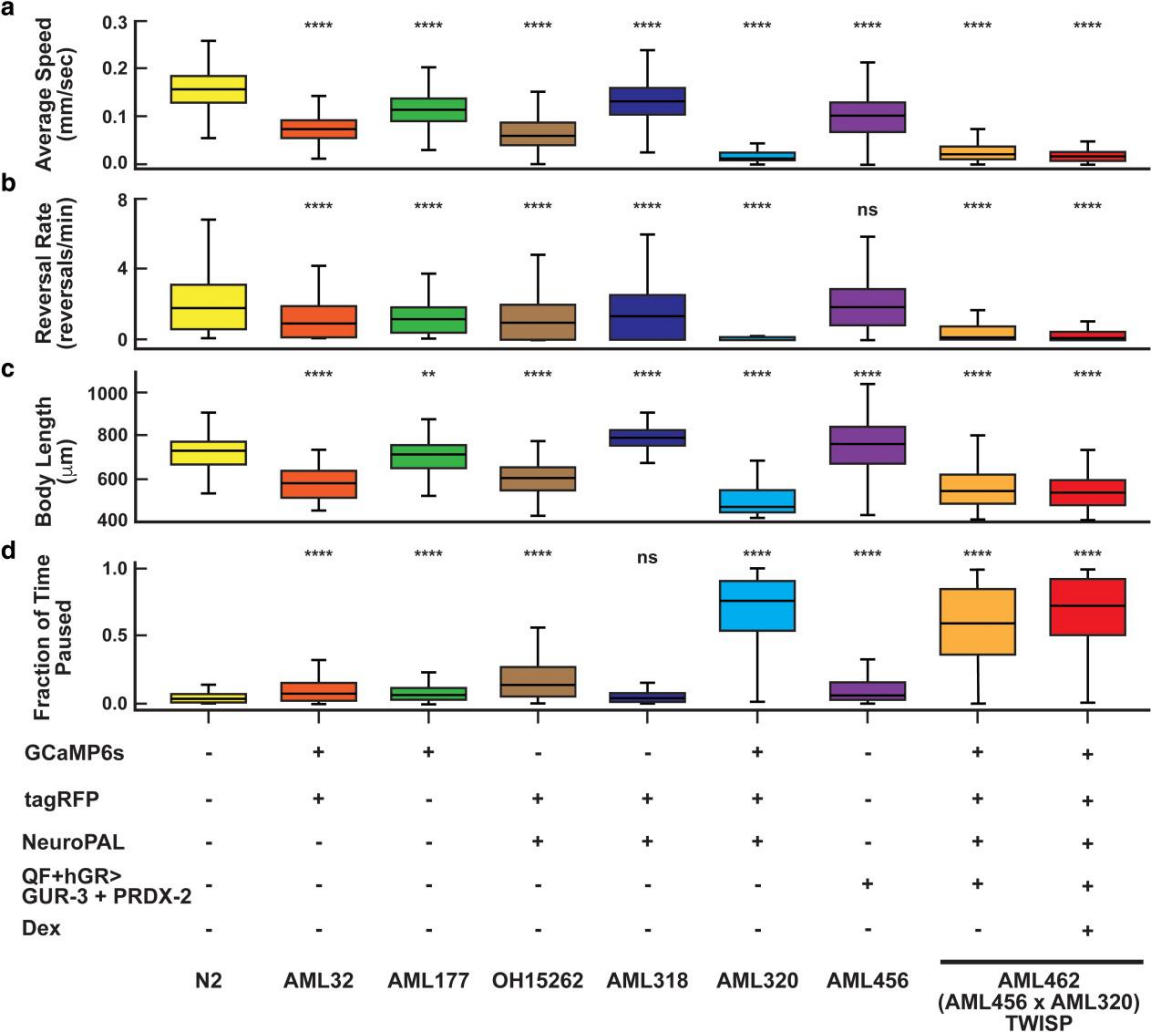
Locomotion decreases with transgenic load, GCaMP6s, NeuroPAL system, and actuator expression. a) Speed, b) reversal rate, c) body length, and d) fraction of time paused are reported for animals from strains containing various components of the TWISP system. The TWISP strain is measured with and without dexamethasone treatment. The combination of NeuroPAL and GCaMP6s expression decreases locomotion. Box and whisker plots report the distribution of behavior across animal tracks (not plates). The number of tracks recorded per condition, from left to right, are N = [1706, 304, 1283, 1232, 1940, 393, 1374, 723, 647]. Box indicates median and interquartile range. Whiskers indicate range excluding outliers. Mean ± SD values and number of plates are reported in Supplementary Table 6. Statistical significance is with respect to WT, using Kruskal–Wallis test followed by Dunn's multiple comparisons, **<0.0021, ****<0.0001.

We tested several actuators (Fig. 1c) for their ability to induce behavioral responses in worms upon illumination with 475 nm or 505 nm light. We were particularly interested in identifying actuators that had robust responses at short wavelength light (e.g. 475 nm) but no response at the longer 505 nm wavelength light we planned to use for imaging GCaMP. We chose 505 nm light because it is still close to GCaMP6s’ excitation peak of 498 nm and is expected to excite GCaMP with similar efficiency to commonly used 488 nm light, despite its longer wavelength (Chen *et al*. 2013). Worms expressing Chrimson or Channelrhodopsin in all neurons with the necessary all-trans retinal co-factor exhibited behavioral responses to 475 and 505 nm illumination, confirming that these opsins suffer from unwanted crosstalk under illumination used for GCaMP6s imaging.

We therefore turned our attention to two optogenetic actuators with blue-shifted excitation spectra relative to Channelrhodopsin: the opsin TsChR (Klapoetke *et al*. 2014; Farhi *et al*. 2019) and a light-sensitive gustatory receptor homolog GUR-3 that is endogenous to the worm (Bhatla and Horvitz 2015). Both are reported to be activated efficiently by wavelengths near 475 nm light, but much less so by 505 nm light (Fig. 1b) (Klapoetke *et al*. 2014; Bhatla and Horvitz 2015) (we note GUR-3's published action spectra is less well characterized than TsChR's). Animals expressing TsChR or GUR-3 in every neuron showed robust behavioral response to 475 nm but little or no response to 505 nm as desired (Fig. 1c), suggesting that TsChR and GUR-3 avoid unwanted activation when imaging GCaMP6s at 505 nm.

In these experiments, animals expressing opsins such as ChR2 or TsChR were tested with and without the necessary co-factor all-trans retinal. Opsins required all-trans retinal to elicit a behavioral response, as expected (Fig. 1c). We also tested GUR-3 with and without co-expression of its associated peroxiredoxin, PRDX-2, thought to improve the efficiency of its light response (Liu *et al*. 2010; Bhatla and Horvitz 2015; Gong *et al*. 2017; Quintin *et al*. 2022). PRDX-2 improved the robustness of the light-induced behavior response (Fig. 1c), therefore in the rest of the manuscript, we always consider the case of GUR-3 co-expressed with PRDX-2. For each condition, proteins were expressed under a pan-neuronal *rab-3* promoter. Both 475 and 505 nm light was delivered at an intensity of ∼1.5 mW/mm^2^. We used a hand-scoring method on a 4-point scale to characterize the animal's light responses. In the highest score, “+++” animals typically coiled within 8 s of illumination. In the next highest score, “++” animals typically paused within 8 s and only later coiled. In the second lowest score, “+” animals paused or reversed but did not coil. And in the lowest score, “−” animals rarely responded at all. Later in this work, we also used a related TsChR variant, eTsChR, that is optimized for more efficient trafficking to the membrane but is otherwise similar (Farhi *et al*. 2019).

Taken together, we concluded that TsChR and GUR-3 + PRDX-2 showed promise for co-expression with GCaMP6s while avoiding optical crosstalk. We therefore sought to generate strains with stable and strong expression.

### Constitutive overexpression of pan-neuronal TsChR or GUR-3 + PRDX-2 is lethal

To be useful for probing neural signaling, any optogenetic actuator must be expressed consistently and at sufficiently high levels. We explored different pan-neuronal expression levels of the actuators by injecting different concentrations of DNA plasmids for either TsChR or GUR-3 + PRDX-2, each with a fluorescent protein (tagBFP or tagRFP, respectively) via co-injectable marker or a SL2 splice site. Higher concentration injections were lethal, but at injection concentrations of 50 ng/µl or below, we were able to generate lines that carried the actuator and fluorescent protein in an extrachromosomal array (Fig. 2a). For example, the 35 ng/µl injection concentration GUR-3 + PRDX-2 showed strong responses (Fig. 1c). We observed variability in expression levels between animals, as expected for extrachromosomal arrays. For TsChR expressing animals, most animals had very dim expression of the co-expressed tagBFP reporter and had long generation time, a potential sign of toxicity. Rarely did we observe worms that had high expression. L1s with high expression never developed into adulthood. By comparing observations of the animal's fluorescent expression with its behavior responses to blue light, we confirmed that higher expression is needed to achieve more robust neural activation. The rare L4s with bright co-expressed BFP responded to 475 nm light by pausing and then coiling (Figs. 2b and 1c), while animals from the same strain with lower expression, which were more common, merely paused.

We tried but failed to use traditional methods to create stable transgenic lines. For example, we attempted to integrate TsChR into the genome using both classical UV (Evans 2006) and miniSOG-assisted blue-light integration (Noma and Jin 2018). Both approaches failed to create stable lines when attempted with 50 ng/µl concentration plasmid injections. This, along with the earlier observation that bright L1-stage extrachromosomal animals never developed to adulthood, both suggest that constitutive overexpression of TsChR may be toxic or prevent development. Therefore, we sought another strategy to generate stable high expression transgenic worms.

We hypothesized that toxicity from actuator overexpressing may be highest early in development and that this may act as a bottleneck preventing high expressing lines from growing to adulthood and propagating. The pan-neuronal *rab-3* promoter that we use (Tursun *et al*. 2011; Nguyen *et al*. 2016; Venkatachalam *et al*. 2016; Yemini *et al*. 2021) drives mRNA expression at levels that peak early in development in L1 and then decreases steadily, driving the lowest levels of expression in adulthood (Davis *et al*. 2022). If the strong expression early in development was toxic, it could explain why we observe bright L1 expression in animals that fail to develop, and why we faced challenges in generating stable integrated lines. We reasoned that turning off expression early in development may allow us to skip over this bottleneck to generate stable transgenic lines with higher expression later in development. We therefore sought to temporally control expression of our optogenetic actuators.

### Temporal control of actuator expression via QF + hGR > QUAS allows for creation of stable lines with inducible high expression

To achieve viable high expression in adulthood, we temporally controlled the expression of the optogenetic actuator using a drug-inducible QF + hGR > QUAS expression system (Monsalve *et al*. 2019). We introduced a heterologous gene expression system (Q system) under the control of an exogenous human glucocorticoid receptor binding site. When the drug dexamethasone (dex) is applied, it activates an engineered protein, QF + hGR, which then binds to the “QUAS” DNA sequence and activates downstream gene expression of the optogenetic actuator.

Injecting plasmids containing this drug-inducible system resulted in viable animals that carried transgenes for the optogenetic actuator under the control of the QF + hGR > QUAS system in an extrachromosomal array (Fig. 3). Injections were viable even for plasmid concentrations of 75–85 ng/µl (Fig. 3a), higher than before (Fig. 2a). The plasmids were designed to express either eTsChR (for AML438) or GUR-3 + PRDX-2 (for AML405) in all neurons via *rab-3* promoter driven QF + hGR upon exposure to dexamethasone (Supplementary Fig. 2 and Supplementary Table 2). We confirmed that the QF + hGR machinery was expressed into adulthood after dex-treatment by testing the animal’s behavior response to 475 nm light (Fig. 3b). We exposed L4 animals to dexamethasone by placing them on NGM plates containing 200 µM dexamethasone overnight.

Animals exposed to dexamethasone exhibited stronger responses to light than similarly aged animals cultivated without dexamethasone (Fig. 3b), indicating that drug-inducible expression was successful. The strength of the response of dexamethasone treated animals was similar to that for the highest-expressing animals under traditional nondrug-inducible expression (compare Fig. 3b to Fig. 2a). Importantly, the vast majority of drug-inducible animals on dex exhibited strong responses, whereas few of the nondrug-inducible animals had strong responses. We therefore conclude that temporal control of this optogenetic actuator allows for consistent strong expression of actuator during adulthood under dex-treatment.

The drug-inducible GUR-3 + PRDX-2 strain showed slightly stronger light-evoked responses than a drug-inducible eTsChR strain (Fig. 3b). We therefore generated a stable integrated strain, AML456, via UV integration, that expresses GUR-3 + PRDX-2 under the drug-inducible system (QF + hGR > GUR-3 + PRDX-2), along with a GFP coelomocyte marker, and outcrossed with N2 eight times.

We used the drug-inducible strain to investigate the role of induced expression of optogenetic proteins on the animal's generation time. Among inducible animals that were not exposed to dexamethasone, the fastest growing ones showed wild-type like development (Fig. 4). Among inducible animals cultivated on dexamethasone for their entire lives, however, even the fastest growing animals showed growth retardation, had notably longer generation times, and visually appeared sick after the second generation (Fig. 4). These animals qualitatively resembled the non-inducible actuator expressing animals (Fig. 2). Interestingly inducible animals cultivated on dexamethasone reverted back to improved health and improved generation times if they were then grown without dexamethasone (Fig. 4). These observations are consistent with our hypothesis that the pan-neuronal expression of the actuator creates a toxicity bottleneck, likely in early stages of developmental. We conclude that the drug-inducible system allows stable transgenic lines to be generated and propagated in a healthy and more timely manner by avoiding this toxicity bottleneck.

### TWISP—a transgenic worm for interrogating signal propagation

To generate a strain for measuring signal propagation, we combined our drug-inducible actuator system with a calcium indicator and with the NeuroPAL multicolor fluorescent system for neuronal identification (Yemini *et al*. 2021). The NeuroPAL system expresses multiple fluorophores combinatorially in each neuron to enable identification of each neuron with respect to the connectome and to other datasets. The system works by converting each neuron's gene expression profile into a genetically encoded fluorescent color code. We crossed our drug-inducible QF + hGR > GUR-3 + PRDX-2 actuator line, AML456, into a NeuroPAL + GCaMP6s line, AML320 (Yu *et al*. 2021) to create TWISP, a transgenic worm for interrogating signal propagation, AML462. TWISP contains around 50 plasmid constructs integrated into the genome. Each neuron expresses GCaMP6s; tagRFP-T; some combination of tagBFP2, cyOFP, and/or mNeptune; and, upon dexamethasone treatment, GUR-3 and PRDX-2. We characterized TWISP's light sensitivity as a function of the duration of the dexamethasone treatment (Supplementary Fig. 4).

We sought to characterize changes to the animal’s vitality and behavior upon the addition of so many genetic comoponents. For example, NeuroPAL strains had previously been reported to be less active (Yemini *et al*. 2021). We therefore measured behavior and lifespan in several transgenic lines (Figs. 5 and 6 and Supplementary Fig. 3). TWISP is noticeably slower, less active, produces animals that have shorter body lengths, has a smaller proportion of animals reach adulthood after 70 h, and produce fewer progeny per unit time than wild-type animals. Notably, TWISP is similar in most of these features to our 14× outcrossed NeuroPAL + GCaMP6s strain, AML320 (Figs. 5 and 6 and Supplementary Fig. 3) (Yu *et al*. 2021), suggesting that the optogenetic actuator may not be the major contributor to these effects.

**Fig. 7.**
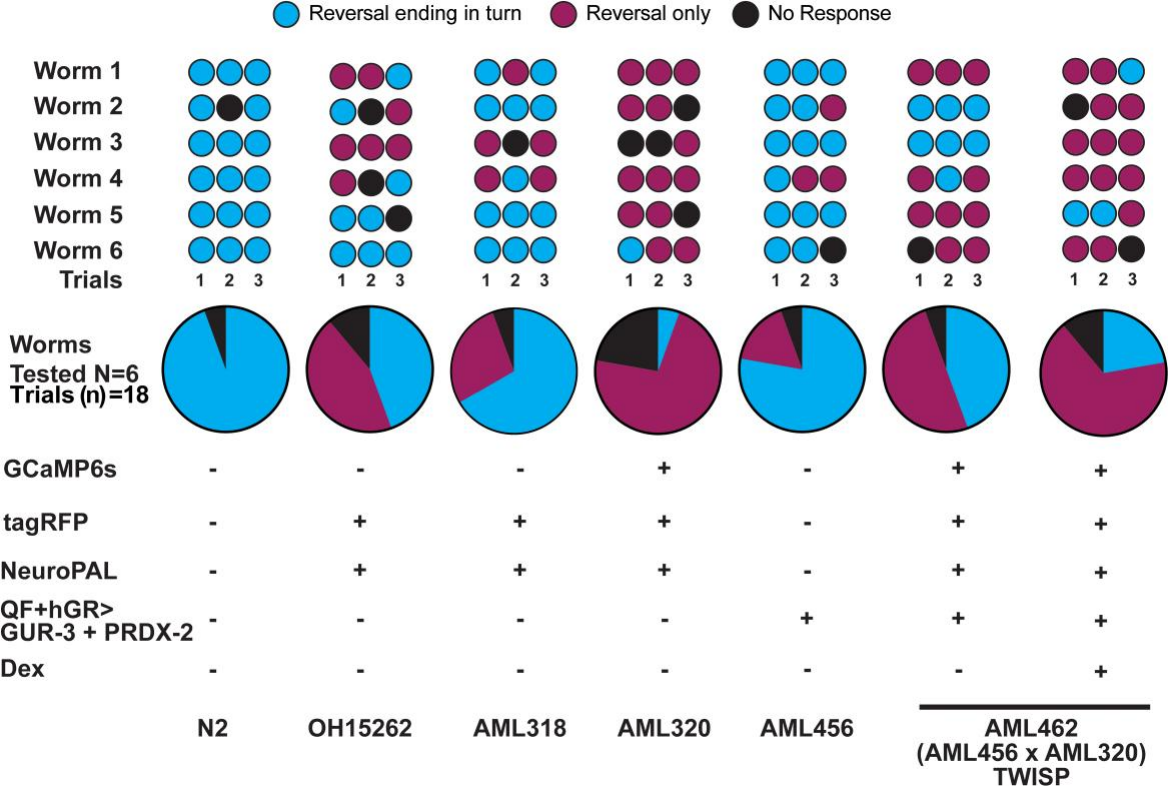
TWISP worms respond to mechanosensory stimuli in a gentle-touch behavior assay. Small circles indicate responses to individual touch stimuli. Six animals were tested for each strain and three stimuli were delivered per animal.

We characterized the animal's behavior response to mechanosensory stimuli using a manual gentle-touch assay (Chalfie *et al*. 2014). TWISP animals maintain a robust touch response (Fig. 7).

### Using TWISP for measuring signal propagation

**Fig. 8.**
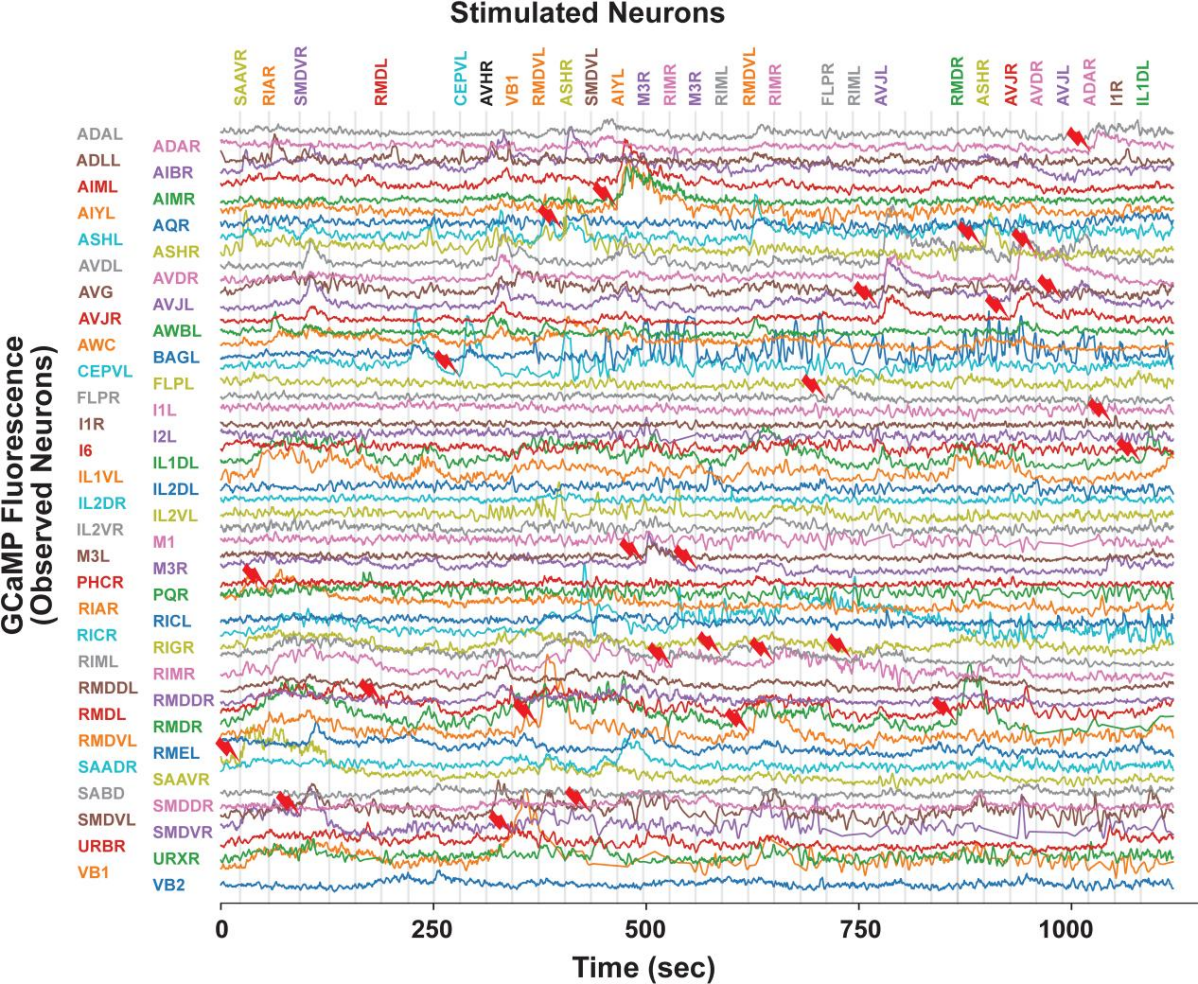
Head and tail population calcium activity in response to neural activation measured with TWISP. Calcium activity of simultaneously recorded neurons from the head and tail is shown during targeted optogenetic stimulation of individual neurons. Calcium imaging is performed via single-photon spinning disk with 505 nm illumination while individual neurons are stimulated via 2-photon spatially restricted illumination at 850 nm. Neuron identities are listed on the left. Gray vertical line indicates times in which a stimulus was delivered (at every 30 s). Red thunderbolt indicates the stimulated neuron. The name of the neuron stimulated is listed above. Recordings of unidentified neurons or of neurons that were not well-segmented are excluded from the plot.

To demonstrate the utility of TWISP for measuring signal propagation among neurons with known neural identities, we stimulated a selection of neurons, one neuron at a time while recording neural population calcium activity (Fig. 8 and Supplementary Fig. 5). Stimulations were performed using spatially restricted 2-photon 850 nm illumination (Randi *et al*. 2023). We typically stimulated a different neuron every 30 s (Fig. 8) or every minute (Supplementary Fig. 5). For example, we stimulated neuron AIYL and observed large calcium responses in AIYL, AIMR, and AIML (Fig. 8). These responses would have been challenging to predict because there are no known direct monosynaptic connections from AIY to AIM. TWISP therefore enables systematic perturbation mapping to investigate how signals propagate through the worm nervous system at brain scale and cellular resolution.

## Discussion

In this work, we presented TWISP, a transgenic worm strain for measuring and manipulating neural activity across the nervous system. The key achievements of this system are (1) spectral separation of actuator and indicator that allows for calcium imaging without unwanted optogenetic activation, (2) stable and robust pan-neuronal expression without lethality, and (3) compatibility with a neural identification system, in this case NeuroPAL (Yemini *et al*. 2021). To our knowledge, no previous system for all-optical neurophysiology has achieved all three of these requirements. Therefore, we believe that TWISP is the first system to allow optical access to activate any *C. elegans* neuron while simultaneously recording from all others, with the knowledge of their identities. TWISP allows for large-scale measurements of how signals propagate through the brain in response to perturbations. In related work, we have used TWISP to measure a comprehensive signal propagation atlas of *C. elegans* (Randi *et al*. 2023), an endeavor that is only made possible by a system like TWISP.

TWISP makes tradeoffs to achieve its robust pan-neuronal expression with minimal spectral overlap. First, to achieve spectral separation it uses an unconventional optogenetic actuator, the GUR-3 + PRDX-2 system (Bhatla and Horvitz 2015), that is less well characterized compared to traditionally used optogenetic proteins, such as Channelrhodopsin. GUR-3 is a *C. elegans* gustatory receptor homolog that sits in the membrane and is thought to respond to reactive oxygen species generated by light via the protein PRDX-2. PRDX-2 is thought to detect reactive oxygen species intracellularly and then activate GUR-3 at the membrane, which in turn is proposed to evoke neural activity via second messengers (Bhatla and Horvitz 2015; Quintin *et al*. 2022). The exact mechanism is unknown, however. In a competing theory it has been proposed that GUR-3 may instead act on the membrane potential directly, possibly as a light-gated ion channel similar to LITE-1 (Hanson *et al*. 2023). Regardless of the mechanism, GUR-3 + PRDX-2 has already been shown to evoke light-dependent calcium transients (Bhatla and Horvitz 2015), to release glutamate to downstream synaptic partners (Bhatla *et al*. 2015), and to evoke behavioral responses (Bhatla and Horvitz 2015; Randi *et al*. 2023), all consistent with its role as an effective light-sensitive neural actuator. Notably, light-evoked GUR-3 dependent calcium transients have similar GCaMP6s fluorescent rise and fall times to those evoked via natural odor stimuli (compare, for example Bhatla and Horvitz (2015) to Lin *et al*. (2023)). A potential advantage of GUR-3 + PRDX-2 compared to a traditional opsin is that GUR-3 + PRDX-2 is activatable by light delivered to the soma, and therefore avoids the need to illuminate the cell membrane. This is advantageous because illuminating intracellularly is more convenient for restricting 2-photon illumination to a single neuron while avoiding its neighbors. Intracellular illumination, for example, avoids the need for shaping a 2-photon excitation spot to match the extended shape of a cell's membrane, which can be challenging especially in larger and irregularly shaped neurons common in mammalian systems.

TWISP trades off the health and vitality of the animal in order to express an actuator, calcium sensor, and neural identification marker in every neuron. The use of a drug-inducible system enables TWISP to be viably maintained and propagated (Fig. 4). But the resulting strain, with neural identity markers, is notably less healthy, as shown in Figs. 5 and 6 and Supplementary Fig. 3, as expected for a strain that expresses ∼50 transgenes. Importantly, the animal still exhibits expected behavioral responses, for example in response to gentle-touch stimuli (Fig. 7). We note that much of the effect on health appears to come from the combined addition of the fluorescent markers used in the NeuroPAL strain and GCaMP6s (Fig. 6). NeuroPAL animals have been reported to be less active (Yemini *et al*. 2021). Despite its non-wild-type behavior, the NeuroPAL strain is rapidly becoming a standard in the field because of the value it provides in identifying neurons (Yemini *et al*. 2021; Atanas *et al*. 2023).

Evidence presented here suggests that the most severe toxicity from pan-neuronal expression of an actuator occurs early in development and can be avoided by only inducing expression in adulthood. Future investigations are needed to better understand the exact mechanism of this toxicity. And similarly, more work is needed to understand the mechanism by which fluorophores for neural identification combined with GCaMP produce non-wild-type behavior.

TWISP should enable several new investigations that were previously inaccessible to the *C. elegans* systems neuroscience community. For example, in Randi *et al*. (2023), we are using TWISP to compare signal propagation in wild-type and *unc-31* mutants in order to explore the role of extrasynaptic signaling in the nervous system at brain scale and cellular resolution and to validate predictions based on recent gene expression (Taylor *et al*. 2021) and peptide-receptor interaction screens (Beets *et al*. 2023; Ripoll-Sanchez *et al*. 2023). Similarly, by leveraging the powerful genetics and mutant libraries of the worm, TWISP should enable investigations into the role of specific transmitters and neuropeptides (Chase and Koelle 2007) and genes (Hobert 2013) to provide a better understanding of the molecular genetic underpinnings of the nervous system.

## Supplementary Material

iyae077_Supplementary_Data

## Data Availability

Raw data and supplementary tables are available at the public repository https://doi.org/10.6084/m9.figshare.23868972.v1. Strains used in this work are being made available through the Caenorhabditis Genetics Center (https://cgc.umn.edu/); plasmids will be made available through Addgene (https://www.addgene.org/). Supplementary Table 1 describes strains used in this study. Supplementary Table 2 contains lists of plasmids generated for this work. Supplemental material available at GENETICS online.

## References

[iyae077-B1] Abdelfattah AS, Valenti R, Zheng J, Wong A; GENIE Project Team; Podgorski K, Koyama M, Kim DS, Schreiter ER. 2020. A general approach to engineer positive-going eFRET voltage indicators. Nat Commun. 11(1):3444. doi:10.1038/s41467-020-17322-1. 32651384 PMC7351947

[iyae077-B2] Abdelfattah AS, Zheng J, Singh A, Huang YC, Reep D, Tsegaye G, Tsang A, Arthur BJ, Rehorova M, Olson CVL, et al 2023. Sensitivity optimization of a rhodopsin-based fluorescent voltage indicator. Neuron. 111(10):1547–1563.e9. doi:10.1016/j.neuron.2023.03.009. 37015225 PMC10280807

[iyae077-B3] Atanas AA, Kim J, Wang Z, Bueno E, Becker M, Kang D, Park J, Kramer TS, Wan FK, Baskoylu S, et al 2023. Brain-wide representations of behavior spanning multiple timescales and states in *C. elegans*. Cell. 186(19):4134–4151.e31. doi:10.1016/j.cell.2023.07.035. 37607537 PMC10836760

[iyae077-B4] Azimi Hashemi N, Bergs ACF, Schuler C, Scheiwe AR, Steuer Costa W, Bach M, Liewald JF, Gottschalk A. 2019. Rhodopsin-based voltage imaging tools for use in muscles and neurons of *Caenorhabditis elegans*. Proc Natl Acad Sci U S A. 116(34):17051–17060. doi:10.1073/pnas.1902443116. 31371514 PMC6708366

[iyae077-B5] Beets I, Zels S, Vandewyer E, Demeulemeester J, Caers J, Baytemur E, Courtney A, Golinelli L, Hasakioğulları İ, Schafer WR, et al 2023. System-wide mapping of peptide-GPCR interactions in *C. elegans*. Cell Rep. 42(9):113058. doi:10.1016/j.celrep.2023.113058. 37656621 PMC7615250

[iyae077-B6] Bergs A, Henss T, Glock C, Nagpal J, Gottschalk A. 2022. Microbial rhodopsin optogenetic tools: application for analyses of synaptic transmission and of neuronal network activity in behavior. Methods Mol Biol. 2468:89–115. doi:10.1007/978-1-0716-2181-3_6. 35320562

[iyae077-B7] Bhatla N, Droste R, Sando SR, Huang A, Horvitz HR. 2015. Distinct neural circuits control rhythm inhibition and spitting by the myogenic pharynx of *C. elegans*. Curr Biol. 25(16):2075–2089. doi:10.1016/j.cub.2015.06.052. 26212880 PMC4546535

[iyae077-B8] Bhatla N, Horvitz HR. 2015. Light and hydrogen peroxide inhibit *C. elegans* feeding through gustatory receptor orthologs and pharyngeal neurons. Neuron. 85(4):804–818. doi:10.1016/j.neuron.2014.12.061. 25640076 PMC4408612

[iyae077-B9] Brenner S . 1974. The genetics of *Caenorhabditis elegans*. Genetics. 77(1):71–94. doi:10.1093/genetics/77.1.71. 4366476 PMC1213120

[iyae077-B10] Chalfie M, Hart AC, Rankin CH, Goodman MB. 2014. Assaying mechanosensation. In: Hobert O, editor. The *C. elegans* Research Community, WormBook. doi:10.1895/wormbook.1.172.1. PMC4448936 25093996

[iyae077-B11] Chase DL, Koelle MR. 2007. Biogenic amine neurotransmitters in *C. elegans*. In: Jorgensen EM and Kaplan JM, editors. The C. elegans Research Community, WormBook. doi:10.1895/wormbook.1.132.1. PMC4781333 18050501

[iyae077-B12] Chen TW, Wardill TJ, Sun Y, Pulver SR, Renninger SL, Baohan A, Schreiter ER, Kerr RA, Orger MB, Jayaraman V, et al 2013. Ultrasensitive fluorescent proteins for imaging neuronal activity. Nature. 499(7458):295–300. doi:10.1038/nature12354. 23868258 PMC3777791

[iyae077-B13] Cook SJ, Jarrell TA, Brittin CA, Wang Y, Bloniarz AE, Yakovlev MA, Nguyen KCQ, Tang LT-H, Bayer EA, Duerr JS, et al 2019. Whole-animal connectomes of both *Caenorhabditis elegans* sexes. Nature. 571(7763):63–71. doi:10.1038/s41586-019-1352-7. 31270481 PMC6889226

[iyae077-B14] Dana H, Mohar B, Sun Y, Narayan S, Gordus A, Hasseman JP, Tsegaye G, Holt GT, Hu A, Walpita D, et al 2016. Sensitive red protein calcium indicators for imaging neural activity. Elife. 5:e12727. doi:10.7554/eLife.12727. 27011354 PMC4846379

[iyae077-B15] Davis P, Zarowiecki M, Arnaboldi V, Becerra A, Cain S, Chan J, Chen WJ, Cho J, da Veiga Beltrame E, Diamantakis S, et al 2022. WormBase in 2022—data, processes, and tools for analyzing *Caenorhabditis elegans*. Genetics. 220(4):iyac003. doi:10.1093/genetics/iyac003. 35134929 PMC8982018

[iyae077-B16] Emiliani V, Cohen AE, Deisseroth K, Hausser M. 2015. All-optical interrogation of neural circuits. J Neurosci. 35(41):13917–13926. doi:10.1523/JNEUROSCI.2916-15.2015. 26468193 PMC4604230

[iyae077-B17] Evans TC . 2006. Transformation and microinjection. In: Ambros V, editor. The *C. elegans* Research Community, WormBook. doi:10.1895/wormbook.1.108.1.

[iyae077-B18] Farhi SL, Parot VJ, Grama A, Yamagata M, Abdelfattah AS, Adam Y, Lou S, Kim JJ, Campbell RE, Cox DD, et al 2019. Wide-area all-optical neurophysiology in acute brain slices. J Neurosci. 39(25):4889–4908. doi:10.1523/JNEUROSCI.0168-19.2019. 30952812 PMC6670252

[iyae077-B19] Franconville R, Beron C, Jayaraman V. 2018. Building a functional connectome of the Drosophila central complex. Elife. 7:e37017. doi:10.7554/eLife.37017. 30124430 PMC6150698

[iyae077-B20] Gong J, Yuan Y, Ward A, Kang L, Zhang B, Wu Z, Peng J, Feng Z, Liu J, Xu XZS. 2017. The *C. elegans* taste receptor homolog LITE-1 is a photoreceptor. Cell. 168(1–2):325. doi:10.1016/j.cell.2016.12.040. 28086096

[iyae077-B21] Guo ZV, Hart AC, Ramanathan S. 2009. Optical interrogation of neural circuits in *Caenorhabditis elegans*. Nat Methods. 6(12):891–896. doi:10.1038/nmeth.1397. 19898486 PMC3108858

[iyae077-B22] Hanson SM, Scholüke J, Liewald J, Sharma R, Ruse C, Engel M, Schüler C, Klaus A, Arghittu S, Baumbach F, et al 2023. Structure-function analysis suggests that the photoreceptor LITE-1 is a light-activated ion channel. Curr Biol. 33(16):3423–3435.e5. doi:10.1016/j.cub.2023.07.008. 37527662

[iyae077-B23] Hobert O . 2013. The neuronal genome of *Caenorhabditis elegans*. In: Jorgensen E, editor. The *C. elegans* Research Community, WormBook. doi:10.1895/wormbook.1.161.1. PMC4781646 24081909

[iyae077-B24] Husson SJ, Gottschalk A, Leifer AM. 2013. Optogenetic manipulation of neural activity in *C. elegans*: from synapse to circuits and behaviour. Biol Cell. 105(6):235–250. doi:10.1111/boc.201200069. 23458457

[iyae077-B25] Kim S, Sharma AK, Vatamaniuk OK. 2018. N-terminal extension and C-terminal domains are required for ABCB6/HMT-1 protein interactions, function in cadmium detoxification, and localization to the endosomal-recycling system in *Caenorhabditis elegans*. Front Physiol. 9:885. doi:10.3389/fphys.2018.00885. 30104978 PMC6077975

[iyae077-B26] Klapoetke NC, Murata Y, Kim SS, Pulver SR, Birdsey-Benson A, Cho YK, Morimoto TK, Chuong AS, Carpenter EJ, Tian Z, et al 2014. Independent optical excitation of distinct neural populations. Nat Methods. 11(3):338–346. doi:10.1038/nmeth.2836. 24509633 PMC3943671

[iyae077-B27] Kumar S, Sharma AK, Tran A, Liu M, Leifer AM. 2023. Inhibitory feedback from the motor circuit gates mechanosensory processing in *Caenorhabditis elegans*. PLoS Biol. 21(9):e3002280. doi:10.1371/journal.pbio.3002280. 37733772 PMC10617738

[iyae077-B28] Lin A, Qin S, Casademunt H, Wu M, Hung W, Cain G, Tan NZ, Valenzuela R, Lesanpezeshki L, Venkatachalam V, et al 2023. Functional imaging and quantification of multineuronal olfactory responses in *C. elegans*. Sci Adv. 9(9):eade1249. doi:10.1126/sciadv.ade1249. 36857454 PMC9977185

[iyae077-B29] Liu J, Ward A, Gao J, Dong Y, Nishio N, Inada H, Kang L, Yu Y, Ma D, Xu T, et al 2010. *C. elegans* phototransduction requires a G protein-dependent cGMP pathway and a taste receptor homolog. Nat Neurosci. 13(6):715–722. doi:10.1038/nn.2540. 20436480 PMC2882063

[iyae077-B30] Liu M, Kumar S, Sharma AK, Leifer AM. 2022. A high-throughput method to deliver targeted optogenetic stimulation to moving *C. elegans* populations. PLoS Biol. 20(1):e3001524. doi:10.1371/journal.pbio.3001524. 35089912 PMC8827482

[iyae077-B31] Liu M, Sharma AK, Shaevitz JW, Leifer AM. 2018. Temporal processing and context dependency in *Caenorhabditis elegans* response to mechanosensation. Elife. 7:e36419. doi:10.7554/eLife.36419. 29943731 PMC6054533

[iyae077-B32] Lu Y, Ahamed T, Mulcahy B, Meng J, Witvliet D, Guan SA, Holmyard D, Hung W, Wen Q, Chisholm A, et al 2022. Extrasynaptic signaling enables an asymmetric juvenile motor circuit to produce symmetric undulation. Curr Biol. 32(21):4631–4644.e5. doi:10.1016/j.cub.2022.09.002. 36182701 PMC9643663

[iyae077-B33] Monsalve GC, Yamamoto KR, Ward JD. 2019. A new tool for inducible gene expression in *Caenorhabditis elegans*. Genetics. 211(2):419–430. doi:10.1534/genetics.118.301705. 30504365 PMC6366904

[iyae077-B34] Nguyen JP, Shipley FB, Linder AN, Plummer GS, Liu M, Setru SU, Shaevitz JW, Leifer AM. 2016. Whole-brain calcium imaging with cellular resolution in freely behaving *Caenorhabditis elegans*. Proc Natl Acad Sci U S A. 113(8):E1074–E1081. doi:10.1073/pnas.1507110112. 26712014 PMC4776509

[iyae077-B35] Noma K, Jin Y. 2018. Rapid integration of multi-copy transgenes using optogenetic mutagenesis in *Caenorhabditis elegans*. G3 (Bethesda). 8(6):2091–2097. doi:10.1534/g3.118.200158. 29691291 PMC5982835

[iyae077-B36] Packer AM, Russell LE, Dalgleish HW, Hausser M. 2015. Simultaneous all-optical manipulation and recording of neural circuit activity with cellular resolution in vivo. Nat Methods. 12(2):140–146. doi:10.1038/nmeth.3217. 25532138 PMC4933203

[iyae077-B37] Quintin S, Aspert T, Ye T, Charvin G. 2022. Distinct mechanisms underlie H_2_O_2_ sensing in *C. elegans* head and tail. PLoS One. 17(9):e0274226. doi:10.1371/journal.pone.0274226. 36173997 PMC9521893

[iyae077-B38] Randi F, Leifer AM. 2020. Measuring and modeling whole-brain neural dynamics in *Caenorhabditis elegans*. Curr Opin Neurobiol. 65:167–175. doi:10.1016/j.conb.2020.11.001. 33279794 PMC7801769

[iyae077-B39] Randi F, Sharma AK, Dvali S, Leifer AM. 2023. Neural signal propagation atlas of *Caenorhabditis elegans*. Nature. 623(7986):406–414. doi:10.1038/s41586-023-06683-4. 37914938 PMC10632145

[iyae077-B40] Rickgauer JP, Deisseroth K, Tank DW. 2014. Simultaneous cellular-resolution optical perturbation and imaging of place cell firing fields. Nat Neurosci. 17(12):1816–1824. doi:10.1038/nn.3866. 25402854 PMC4459599

[iyae077-B41] Ripoll-Sanchez L, Watteyne J, Sun H, Fernandez R, Taylor SR, Weinreb A, Bentley BL, Hammarlund M, Miller DM, Hobert O, et al 2023. The neuropeptidergic connectome of *C. elegans*. Neuron. 111(22):3570–3589.e5. doi:10.1016/j.neuron.2023.09.043. 37935195 PMC7615469

[iyae077-B42] Simpson JH, Looger LL. 2018. Functional imaging and optogenetics in Drosophila. Genetics. 208(4):1291–1309. doi:10.1534/genetics.117.300228. 29618589 PMC5887132

[iyae077-B43] Smith SJ, Sumbul U, Graybuck LT, Collman F, Seshamani S, Gala R, Gliko O, Elabbady L, Miller JA, Bakken TE, et al 2019. Single-cell transcriptomic evidence for dense intracortical neuropeptide networks. Elife. 8:e47889. doi:10.7554/eLife.47889. 31710287 PMC6881117

[iyae077-B44] Taylor SR, Santpere G, Weinreb A, Barrett A, Reilly MB, Xu C, Varol E, Oikonomou P, Glenwinkel L, McWhirter R, et al 2021. Molecular topography of an entire nervous system. Cell. 184(16):4329–4347.e3. doi:10.1016/j.cell.2021.06.023. 34237253 PMC8710130

[iyae077-B45] Tursun B, Patel T, Kratsios P, Hobert O. 2011. Direct conversion of *C. elegans* germ cells into specific neuron types. Science. 331(6015):304–308. doi:10.1126/science.1199082. 21148348 PMC3250927

[iyae077-B46] Venkatachalam V, Ji N, Wang X, Clark C, Mitchell JK, Klein M, Tabone CJ, Florman J, Ji H, Greenwood J, et al 2016. Pan-neuronal imaging in roaming *Caenorhabditis elegans*. Proc Natl Acad Sci U S A. 113(8):E1082–E1088. doi:10.1073/pnas.1507109113. 26711989 PMC4776525

[iyae077-B47] White JG, Southgate E, Thomson JN, Brenner S. 1986. The structure of the nervous system of the nematode *Caenorhabditis elegans*. Philos Trans R Soc Lond B Biol Sci. 314(1165):1–340. doi:10.1098/rstb.1986.0056. 22462104

[iyae077-B48] Witvliet D, Mulcahy B, Mitchell JK, Meirovitch Y, Berger DR, Wu Y, Liu Y, Koh WX, Parvathala R, Holmyard D, et al 2021. Connectomes across development reveal principles of brain maturation. Nature. 596(7871):257–261. doi:10.1038/s41586-021-03778-8. 34349261 PMC8756380

[iyae077-B49] Yemini E, Lin A, Nejatbakhsh A, Varol E, Sun R, Mena GE, Samuel ADT, Paninski L, Venkatachalam V, Hobert O. 2021. NeuroPAL: a multicolor atlas for whole-brain neuronal identification in *C. elegans*. Cell. 184(1):272–288.e11. doi:10.1016/j.cell.2020.12.012. 33378642 PMC10494711

[iyae077-B50] Yu X, Creamer MS, Randi F, Sharma AK, Linderman SW, Leifer AM. 2021. Fast deep neural correspondence for tracking and identifying neurons in *C. elegans* using semi-synthetic training. Elife. 10:e66410. doi:10.7554/eLife.66410. 34259623 PMC8367385

