# Supplementary material for: TWISP: a transgenic worm for interrogating signal propagation in Caenorhabditis elegans
Source: Genetics. 2024 May 11;227(3):iyae077. doi: 10.1093/genetics/iyae077 (PMC11228852; doi:10.1093/genetics/iyae077)
Supplement: iyae077_Supplementary_Data [file iyae077_supplementary_data.docx]

Supplementary Data: Sharma et al., 2024.

**Title:** TWISP: A Transgenic Worm for Interrogating Signal Propagation in *C. elegans*

**Authors:**

Anuj Kumar Sharma^1^, Francesco Randi^1, 3, †^, Sandeep Kumar^2^, Sophie Dvali^1^ and Andrew Leifer^1, 2^ **^*^**

**Affiliation:**

**^1^ Department of Physics, Princeton University, Princeton, NJ, 08544**

**^2^ Princeton Neuroscience Institute, Princeton University, Princeton, NJ, 08544**

**^3^Regeneron Pharmaceutical Inc.**

† **Current address: Regeneron Pharmaceuticals Inc.,** FR contributed to this article as an employee of Princeton University and the views expressed do not necessarily represent the views of Regeneron Pharmaceuticals Inc.

**^*^ Corresponding Author:** Department of Physics and Princeton Neuroscience Institute, Princeton University, Princeton, New Jersey, 08544  Email: **leifer@princeton.edu**

**Supplementary Figure S1:** Spectra of blue light used in behavior response assays performed in Fig. 1c, Fig. 2b and Fig. 3b.


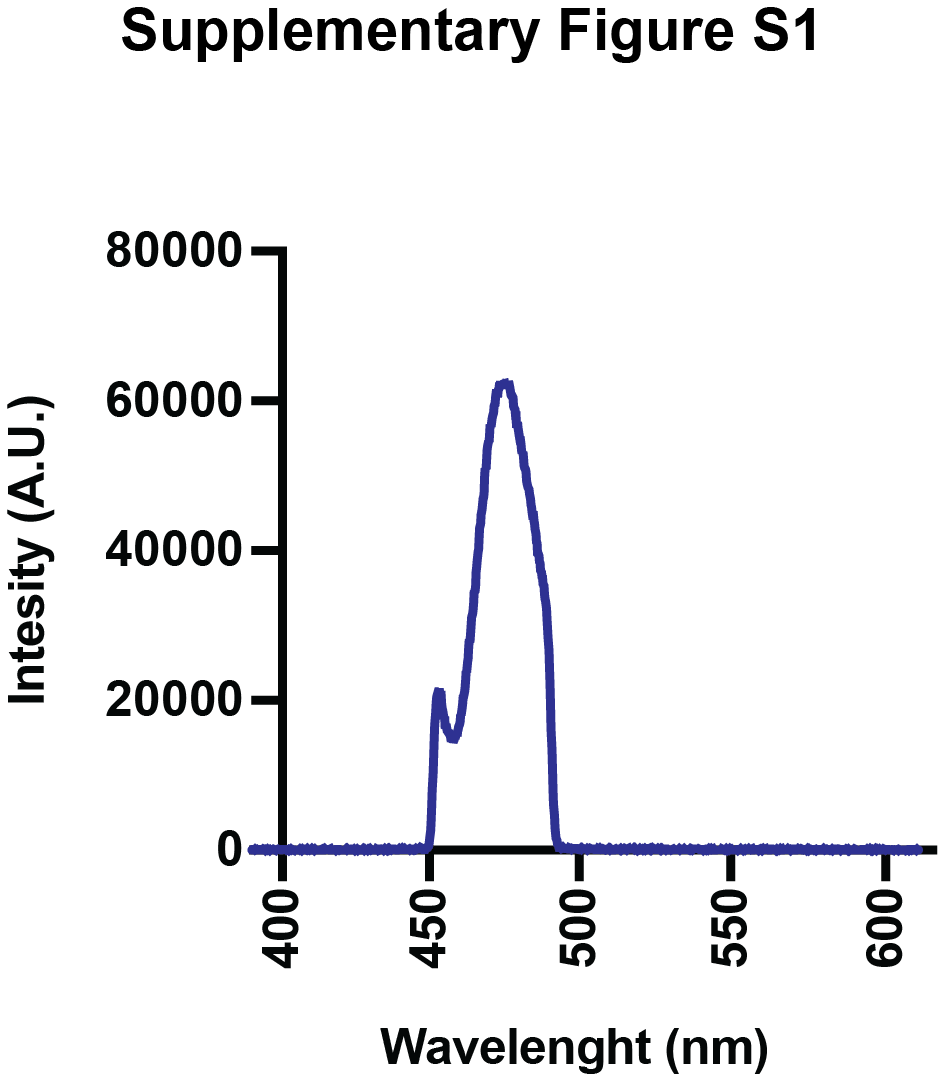


**Supplementary Figure S2:** Plasmid vectors designed for Dex inducible (QF+hGR>QUAS) expression of a.) eTsChR and b.) GUR-3 + PRDX-2.


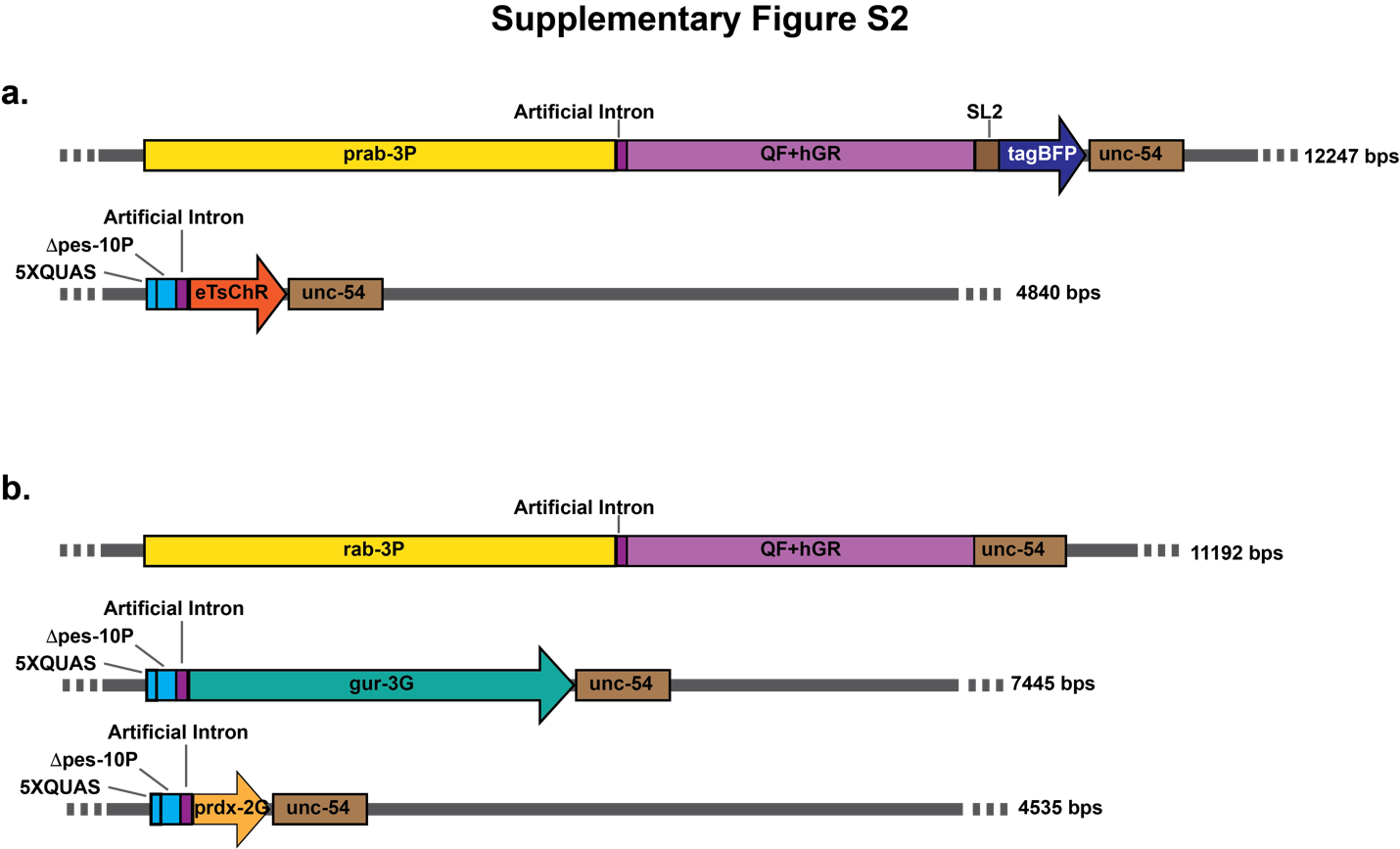


**Supplementary Figure S3:** Example locomotory trajectories transgenic animals.Representative traces are selected from hundreds to thousands of trajectories measured for each strain. The five example tracks for each strain were chosen by selecting the median-length track, and the four nearest length tracks (two shorter, two longer), from a ranked list of the tracks in each condition, ranked by the track length in seconds. Tracks are displayed such that the midpoint of their track is at the origin. Colorbar indicates the time that has elapsed since the beginning of the track.


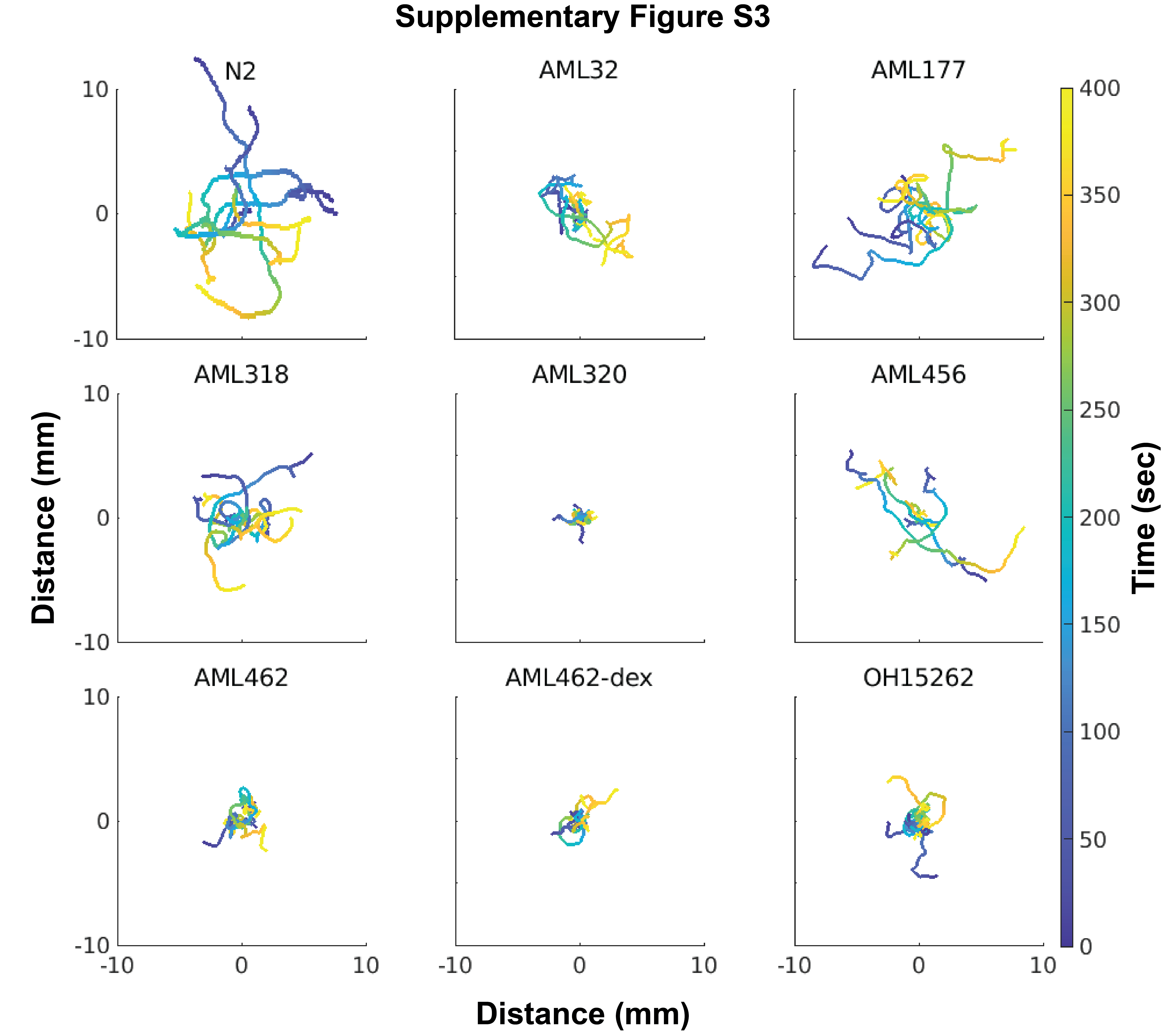


**Supplementary Figure S4:** Effect of the duration of dexamethasone-treatment on TWISP’s light response. Worms were subjected to dexamethasone treatment starting at an age that ranged from L3 to young adult, depending on the treatment duration. Response to light was always evaluated at the end of treatment in early adulthood.


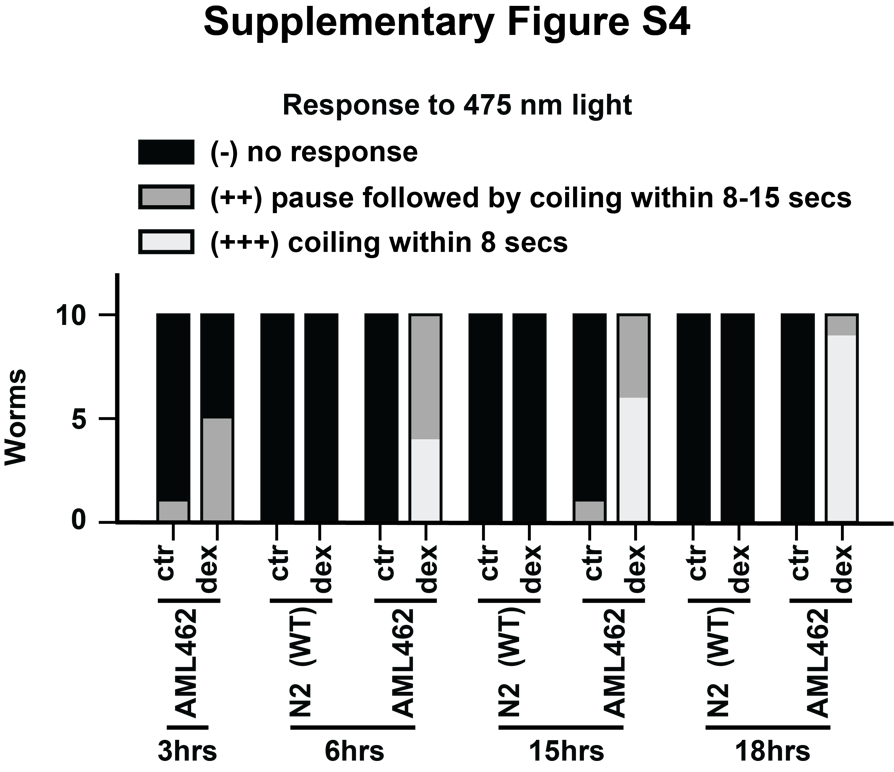


**Supplementary Figure S5:** Calcium activity of simultaneously recorded neurons from the head shown during targeted optogenetic stimulation of individual neurons. Calcium imaging is performed via single photon spinning disk with 505nm illumination while individual neurons are stimulated via two photon spatially restricted illumination at 840nm. Neuron identities are listed on the left. Gray vertical line indicates times in which a stimulus was delivered (every minute in this case). Red thunderbolt indicates the stimulated neuron. The name of the neuron stimulated is listed above. Recordings of unidentified neurons or of neurons that were not well-segmented are excluded from the plot.


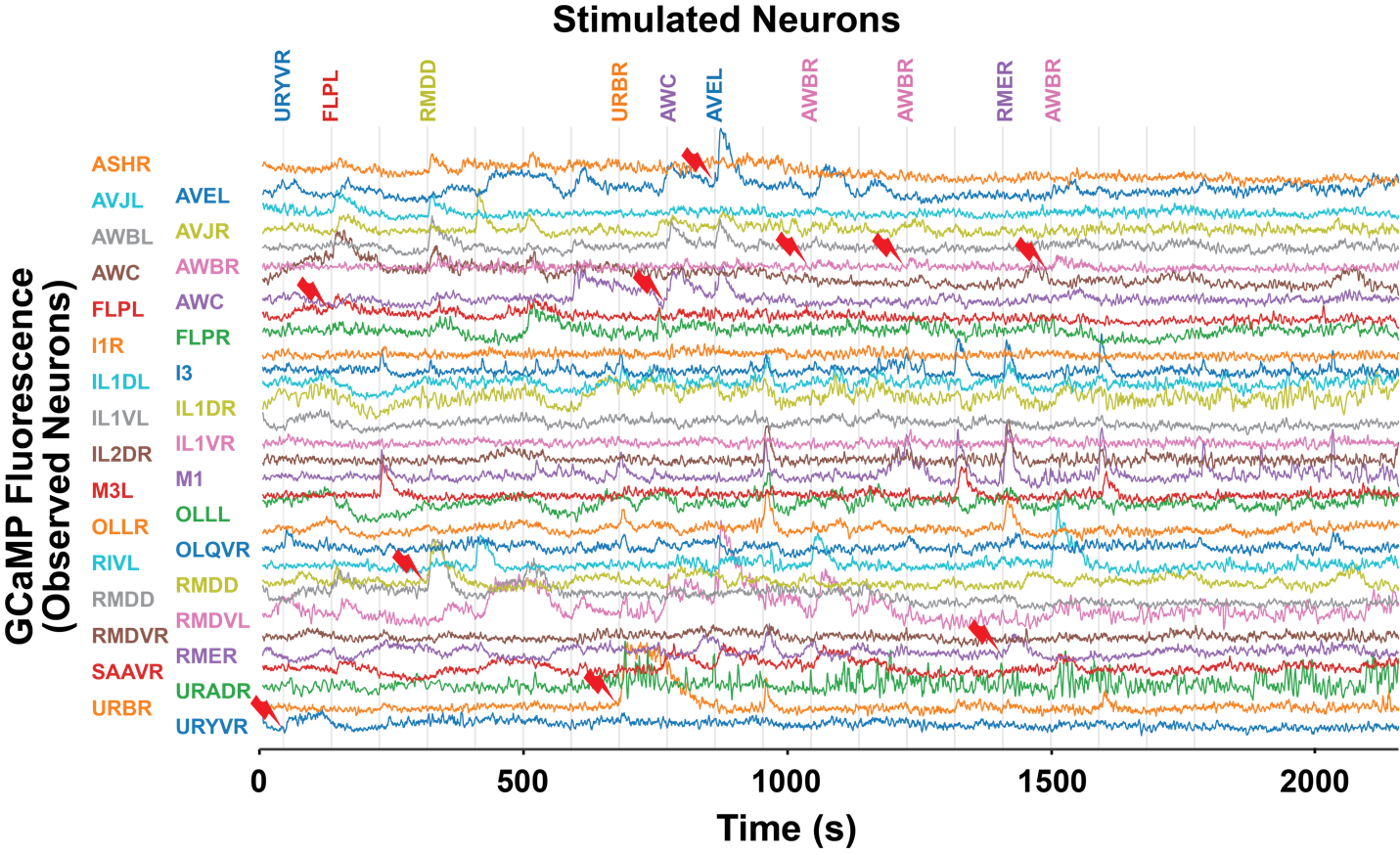


**Supplementary Table S1:** List of worms used, and transgenic worms created for this work, sorted by figure in which they first appear.

| **Strain** | **Genotype** | **Expression/ Identifier** | **Role** | **Reference** | **Used in Figure** |
| --- | --- | --- | --- | --- | --- |
| **N2** | Wild-type | - | control | - | Fig.1, 4, 5, 6 and 7  Supplementary Fig. S3 and S4  Supplementary Tables S3, S4, S5, S6 |
| **AML344** | *juSi164 unc-119(ed3) III; wtfIs263 [rab-3P::AI::ChR2(H134R)::unc-54 35 ng/µl + rab-3P::AI::Voltron::unc-54 70 ng/µl + rab-3P::his-24::tagBFP::unc-54 30 ng/µl]* | Pan-neuronal-ChR2 (H134R) | Testing ChR2 response | In this study | Fig. 1 |
| **AML73** | *pha-1(e2123)III; wtfEX48 [pBX 30ng/µl + rab-3P::CHRIMSON_CE::unc-54 30ng/µl]* | Pan-neuronal-Chrimson | Testing Chrimson response | In this study | Fig. 1 |
| **AML188** | *wtfEx157 [rab-3P::his-24::tagBFP::unc-54 50 ng/µl+ rab-3P::AI::TsChR::unc-54 30 ng/µl]* | Pan-neuronal TsChR | Testing TsChR response | In this study | Fig.1, 2 and 3a |
| **AML532** | *wtfEx475 [rab-3P::AI::gur-3G::SL2::tagBFP::unc-54 70ng/µl + unc-122P::GFP 80ng/µl]* | Pan-neuronal GUR-3/ pan-neuronal tagBFP/Coel::GFP | Testing GUR-3 response | In this study | Fig. 1 |
| **AML376** | *juSi164 unc-119(ed3) III; wtfEx296 [rab-3P::AI::gur-3G::unc-54 35 ng/µl + rab-3P::AI::prdx-2G::SL2::his-24::tagRFP::unc-54 35 ng/µl + rab-3P::his-24::GCaMP6s::unc-54 100 ng/µl]* | Pan-neuronal GUR-3 + PRDX-2/ nuclear localized, pan-neuronal tagRFP/ nuclear localized, pan-neuronal GCaMP6s | Testing GUR-3 + PRDX-2 response | In this study | Fig. 1, 2a and 3a |
| **AML535** | *wtfEx478 [rab-3P::AI::lite-1G::SL2::tagBFP::unc-54 70 ng/ µl + unc-122P::GFP 80 ng/µl]* | Pan-neuronal LITE-1/ pan-neuronal tagBFP/Coel::GFP | Testing LITE-1 response | In this study | Fig. 1 |
| **AML540** | *juSi164 unc-119(ed3) III; wtfIs483 [rab-3P::AI::lite-1G::unc-54 50 ng/µl + rab-3P::AI::prdx-2G::SL2::his-24::tagRFP::unc-54 30 ng/µl + rab-3P::his-24::GCaMP6s::unc-54 80 ng/µl]* | Pan-neuronal LITE-1+PRDX-2/ nuclear localized, pan-neuronal tagRFP/ nuclear localized, pan-neuronal GCaMP6s | Testing LITE-1+PRDX-2 response |  | Fig. 1 |
| **AML438** | *juSi164 unc-119(ed3) III; wtfIs335 [5xQUAS+Δpes-10P::AI::eTsChR::unc-54 85 ng/µl + rab-3P::AI::QF+hGR::SL2::tagBFP::unc-54 100 ng/µl + rab-3P::his-24::tagRFP::unc-54 25 ng/µl + rab-3P::his-24::GCaMP6s::unc-54 100 ng/µl]* | Pan-neuronal eTsChR on activation using dex treatment via QF+hGR | Testing eTsChR response | In this study | Fig. 3 |
| **AML405** | *juSi164 unc-119(ed3) III; wtfEx315 [5xQUAS::Δpes-10P::AI::gur-3G::unc-54 75 ng/µl + 5xQUAS::Δpes-10P::AI::prdx-2G::unc-54 75 ng/µl + rab-3P::AI::QF+hGR::unc-54 35 ng/µl + unc-122P::GFP 100 ng µl]* | Pan-neuronal QF+hGR>(GUR-3 + PRDX-2)/Coel::GFP | Testing GUR-3 + PRDX-2 response | In this study | Fig. 3 |
| **AML456** | *wtfIs348 [5xQUAS::Δpes-10P::AI::gur-3G::unc-54 75 ng/µl + 5xQUAS::Δpes-10P::AI::prdx-2G::unc-54 75 ng/µl + rab-3P::AI::QF+hGR::unc-54 35 ng/µl + unc-122P::GFP 100 ng µl]* | Pan-neuronal QF+hGR>(GUR-3 + PRDX-2)/Coel::GFP | Testing GUR-3 + PRDX-2 response | In this study | Fig. 4, 5, 6 and 7  Supplementary Fig. S3  Supplementary Tables S3, S4, S5 and S6 |
| **AML462 (TWISP)** | *otIs669[NeuroPAL] V 14x outcrossed; wtfIs145 [pBX 30 ng/µl + rab-3P::his-24::GCaMP6s::unc-54 30 ng/µl]; wtfIs348 [5xQUAS::Δpes-10P::AI::gur-3G::unc-54 75 ng/µl + 5xQUAS::Δpes-10P::AI::prdx-2G::unc-54 75 ng/µl + -rab-3P::AI::QF+hGR::unc-54 35 ng/µl + unc-122P::GFP 100 ng/µl]* | NeuroPAL/ pan-neuronal nuclear localized GCaMP6s /Pan-neuronal QF+hGR>(GUR-3 + PRDX-2)/ Coel::GFP | Recording Signal Propagation/ Neuron Identities | In this study | Fig. 4, 5, 6, 7 and 8  Supplementary Fig. S3, S4 and S5  Supplementary Tables S3, S4, S5 and S6 |
| **AML177** | *wtfIs145 [pBX 30 ng/µl + rab-3P::his-24::GCaMP6s::unc-54 30 ng/µl]* | Nuclear localized, pan-neuronal GCaMP6s | Recording Calcium activity | (Yu *et al.* 2021) | Fig. 5 and 6  Supplementary Fig. S3  Supplementary Tables S3, S4, S5 and S6 |
| **AML32** | *wtfIs5[rab-3P::NLS::GCaMP6s; rab-3P::NLS::tagRFP]* | Pan-neuronal GCaMP6s/tagRFP | Recording Calcium activity | (Nguyen *et al.* 2017) | Fig. 5 and 6  Supplementary Fig. S3  Supplementary Tables S3, S4, S5 and S6 |
| **AML318** | *otIs669[NeuroPAL] V 14x outcrossed* | NeuroPAL | Neuron Identities | In this study | Fig. 5, 6 and 7  Supplementary Fig. S3  Supplementary Table S6 |
| **AML320** | *otIs669[NeuroPAL] V 14x outcrossed; wtfIs145 [pBX 30 ng/µl + rab-3P::his-24::GCaMP6s::unc-54 30 ng/µl]* | NeuroPAL/ pan-neuronal nuclear localized GCaMP6s | Recording Calcium activity/ Neuron Identities | (Yu *et al.* 2021) | Fig. 5, 6 and 7  Supplementary Fig. S3  Supplementary Tables S3, S4 S5, and S6 |
| **OH15262** | *otIs669[NeuroPAL] V 8x outcrossed* | NeuroPAL | Neuron Identities | (Yemini *et al.* 2021) | Fig. 6 and 7  Supplementary Fig. S3  Supplementary Table S6 |
| **CZ20310** | *juSi164 unc-119(ed3) III* | Mini-SOG expression in germline | Creating transgenic worms | (Noma and Jin 2018) |  |

**Supplementary Table S2:** List of plasmids created for this study.

| **Plasmid Created for this study** | **Description** |
| --- | --- |
| *pAS1-rab-3P::his-24::tagRFP* | Pan-neuronal expression of nuclear localized fluorophore “tagRFP”, regulated by *rab-3* promoter and *unc-54* 3' utr |
| *pAS1-rab-3P::his-24::tagBFP* | Pan-neuronal expression of nuclear localized fluorophore “tagBFP”, regulated by *rab-3* promoter and *unc-54* 3' utr |
| *pAS1-rab-3P::his-24::GCaMP6s* | Pan-neuronal expression of nuclear localized calcium sensor “GCaMP6s”, regulated by *rab-3* promoter and *unc-54* 3' utr |
| *pAS3-rab-3P::AI::ChR2(H134R)* | Pan-neuronal expression of opsin “ChR2(H134R)”, regulated by *rab-3* promoter and *unc-54* 3' utr |
| *pAS3-rab-3P::AI::Voltron* | Pan-neuronal expression of voltage sensor “Voltron”, regulated by *rab-3* promoter and *unc-54* 3' utr |
| *pAS3-rab-3P::CHRIMSON_CE* | Pan-neuronal expression of opsin “Chrimson”, regulated by *rab-3* promoter and *unc-54* 3' utr |
| *pAS3-rab-3P::AI::TsChR* | Pan-neuronal expression of opsin “TsChR”, regulated by *rab-3* promoter and *unc-54* 3' utr |
| *pAS3-rab-3P::AI::gur-3G* | Pan-neuronal expression of “GUR-3” using a genomic fragment, regulated by *rab-3* promoter and *unc-54* 3' utr |
| *pAS3-rab-3P::AI::lite-1G* | Pan-neuronal expression of “LITE-1” using a genomic fragment, regulated by *rab-3* promoter and *unc-54* 3' utr |
| *pAS3-rab-3P::AI::QF+hGR::SL2::tagBFP* | Pan-neuronal expression of chimeric protein “QF+hGR” and fluorophore “tagBFP”, regulated by *rab-3* promoter and *unc-54* 3' utr |
| *pAS3-rab-3P::AI::gur-3G::SL2::tagBFP* | Pan-neuronal expression of “GUR-3” using a genomic fragment and fluorophore “tagBFP”, regulated by *rab-3* promoter and *unc-54* 3' utr |
| *pAS3-rab-3P::AI::prdx-2G::SL2::his-24::tagRFP* | Pan-neuronal expression of “PRDX-2” using a genomic fragment and fluorophore “tagRFP”, regulated by *rab-3* promoter and *unc-54* 3' utr |
| *pAS3-rab-3P::AI::lite-1G::SL2::tagBFP::unc-54* | Pan-neuronal expression of “LITE-1” using a genomic fragment and fluorophore “tagBFP”, regulated by *rab-3* promoter and *unc-54* 3' utr |
| *pAS3-rab-3P::AI::QF+hGR* | Pan-neuronal expression of chimeric protein “QF+hGR”, regulated by *rab-3* promoter and *unc-54* 3' utr |
| *pAS3-5xQUAS+Δpes-10P::AI::eTsChR* | Expression of opsin “eTsChR” on activation via “QF+hGR” chimeric protein |
| *pAS3-5xQUAS::Δpes-10P::AI::gur-3G* | Expression of “GUR-3” on activation via “QF+hGR” chimeric protein |
| *pAS3-5xQUAS::Δpes-10P::AI::prdx-2G* | Expression of “PRDX-2” on activation via “QF+hGR” chimeric protein |

**Note:** pAS1 and pAS3 plasmid backbones are including *unc-54* 3' utr sequence.

**Supplementary Table S3:** Data from progeny production assay, corresponding to Figure 5a, including number of plates, total number of worms counted and statistical values.

| **Strain Name (transgene expression)** | **Progeny produced/hr/worm. Mean across plates (+/- SD)** | **pValue** | **Total number of worms counted (n)** | **Number of plates analyzed (N)** |
| --- | --- | --- | --- | --- |
| N2 | 4.68 (2.89) |  | 946 | 24 |
| AML32 (GCaMP6s and tagRFP) | 2.89 (0.68) | >0.999 | 258 | 11 |
| AML177 (GCaMP6s) | 3.36 (1.76) | >0.999 | 339 | 12 |
| AML318 (NeuroPAL) | 3.05 (1.43) | >0.999 | 412 | 15 |
| AML320 (GCaMP6s, tagRFP & NeuroPAL) | 2.40 (1.24) | 0.0117 | 487 | 24 |
| AML456 (GUR-3 + PRDX-2) | 2.40 (1.31) | 0.0129 | 507 | 23 |
| AML462 (GCaMP6s, tagRFP, GUR-3 + PRDX-2 & NeuroPAL) | 2.12 (0.76) | 0.0023 | 439 | 24 |

**Supplementary Table S4:** Data from growth assay at 70 hrs, corresponding to Figure 5b, including number of plates, total number of worms counted and statistical values.

| **Strain Name (transgene expression)** | **Percentage of worms reached to Adulthood at ~70 hrs: Mean across plates (+/- SD)** | **pValue** | **Total number of worms counted (n)** | **Number of plates analyzed** | **Remarks** |
| --- | --- | --- | --- | --- | --- |
| N2 | 99.2 (2.53) |  | 946 | 27 | All adults and very few eggs |
| AML32 (GCaMP6s and tagRFP) | 80.05 (4.99) | 0.0017 | 258 | 11 | Adults and L4s |
| AML177 (GCaMP6s) | 86.56 (9.16) | 0.0437 | 339 | 12 | Adults and L4s |
| AML318 | 94.72 (4.37) | >0.9999 | 412 | 15 | Adults and L4s |
| AML320 (GCaMP6s, tagRFP & NeuroPAL) | 45.23 (42.99) | <0.0001 | 487 | 24 | Populations varies from L2s-L4s and few yAds |
| AML456 (GUR-3 + PRDX-2) | 39.88 (36.90) | <0.0001 | 507 | 23 | Most worms grew up to L3, few L4s and few yAds |
| AML462 (GCaMP6s, tagRFP, GUR-3 + PRDX-2 & NeuroPAL) | 19.38 (17.72) | <0.0001 | 439 | 24 | Populations varies from L2s-L4s and few yAds |

| **Strain Name (transgene expression)** | **Percentage of worms reached to Adulthood at ~94 hrs: Mean across plates (+/- SD)** | **pValue** | **Total number of worms counted (n)** | **Number of plates analyzed** | **Remarks** |
| --- | --- | --- | --- | --- | --- |
| N2 | 100 (0) |  | 629 | 17 | All Adults |
| AML32 (GCaMP6s and tagRFP) | 100 (0) | >0.9999 | 84 | 4 | All Adults |
| AML177 (GCaMP6s) | 100 (0) | >0.9999 | 80 | 5 | All Adults |
| AML318 | 99.46 (1.26) | >0.9999 | 411 | 12 | Mostly Adults |
| AML320 (GCaMP6s, tagRFP & NeuroPAL) | 94.11 (11.38) | 0.3959 | 417 | 17 | Adults and L4s |
| AML456 (GUR-3 + PRDX-2) | 97.98 (4.73) | >0.9999 | 444 | 17 | Adults and L4s |
| AML462 (GCaMP6s, tagRFP, GUR-3 + PRDX-2 & NeuroPAL) | 88.44 (19.24) | 0.0025 | 360 | 17 | Adults with L4s and few L3s |

**Supplementary Table S5:** Data from growth assay at ~94 hrs, corresponding to Figure 5c, including number of plates, total number of worms counted and statistical values.

**Supplementary Table S6:** Data from behavior analysis, corresponding to Figure 6, including number of plates recorded, total number of tracks in analysis and statistical values for each strain for each parameter.

| **Strain Name (transgene expression)** | **Average Speed (mm/sec) Mean (+/- SD), pValue** | **Reversal Rate, (reversals/min) Mean (+/- SD), pValue** | **Length (µm) Mean (+/- SD), pValue** | **Fraction of Time Paused. Mean (+/- SD), pValue** | **Number of Tracks analyzed** | **Number of assays**  **(plates)** |
| --- | --- | --- | --- | --- | --- | --- |
| N2 | 0.15 (0.042) | 2 (1.6) | 711.53 (95.9) | 0.064 (0.12) | 1706 | 12 |
| AML32 (GCaMP6s and tagRFP) | 0.073 (0.023), <0.0001 | 1.2 (1.2), <0.0001 | 579.06 (65.3), <0.0001 | 0.11 (0.13), <0.0001 | 304 | 4 |
| AML177 (GCaMP6s) | 0.11 (0.032), <0.0001 | 1.18 (0.96), <0.0001 | 698.39 (73.4), 0.0036 | 0.084 (0.077), <0.0001 | 1283 | 8 |
| OH15262 ( tagRFP & NeuroPAL) | 0.066 (0.03), <0.0001 | 1.27 (1.46), <0.0001 | 597.42 (71.1), <0.0001 | 0.20 (0.21), <0.0001 | 1232 | 4 |
| AML318 (tagRFP & NeuroPAL) | 0.13 (0.041), <0.0001 | 1.59 (1.55), <0.0001 | 783.84 (57.8), <0.0001 | 0.061 (0.072), 0.0850 | 1940 | 4 |
| AML320 (GCaMP6s, tagRFP & NeuroPAL) | 0.017 (0.011), <0.0001 | 0.17 (0.56), <0.0001 | 503.79 (82.5), <0.0001 | 0.7 (0.24), <0.0001 | 393 | 4 |
| AML456 (GUR-3 + PRDX-2) | 0.097 (0.043), <0.0001 | 1.9 (1.6), 0.9999 | 752.55 (125.0), <0.0001 | 0.15 (0.22), <0.0001 | 1374 | 4 |
| AML462 (GCaMP6s, tagRFP, GUR-3 + PRDX-2 & NeuroPAL) | 0.026 (0.021), <0.0001 | 0.52 (1), <0.0001 | 559.82 (91.0), <0.0001 | 0.59 (0.29), <0.0001 | 723 | 8 |
| AML462 (on-dex) | 0.019 (0.013), <0.0001 | 0.46 (1), <0.0001 | 543.16 (87.5), <0.0001 | 0.69 (0.25), <0.0001 | 647 | 4 |

**Supplementary References:**

Nguyen, J. P., A. N. Linder, G. S. Plummer, J. W. Shaevitz and A. M. Leifer, 2017 Automatically tracking neurons in a moving and deforming brain. PLoS Comput Biol 13**:** e1005517.

Noma, K., and Y. Jin, 2018 Rapid Integration of Multi-copy Transgenes Using Optogenetic Mutagenesis in Caenorhabditis elegans. G3 (Bethesda) 8**:** 2091-2097.

Yemini, E., A. Lin, A. Nejatbakhsh, E. Varol, R. Sun *et al.*, 2021 NeuroPAL: A Multicolor Atlas for Whole-Brain Neuronal Identification in C. elegans. Cell 184**:** 272-288 e211.

Yu, X., M. S. Creamer, F. Randi, A. K. Sharma, S. W. Linderman *et al.*, 2021 Fast deep neural correspondence for tracking and identifying neurons in C. elegans using semi-synthetic training. Elife 10.
